# Differential Regulation of Smad3 and of the Type II Transforming Growth Factor-β Receptor in Mitosis: Implications for Signaling

**DOI:** 10.1371/journal.pone.0043459

**Published:** 2012-08-22

**Authors:** Tal Hirschhorn, Lior Barizilay, Nechama I. Smorodinsky, Marcelo Ehrlich

**Affiliations:** 1 Department of Cell Research and Immunology, George S. Wise Faculty of Life Sciences, Tel Aviv University, Tel Aviv, Israel; 2 The Alec and Myra Marmot Hybridoma Unit, George S. Wise Faculty of Life Sciences, Tel Aviv University, Tel Aviv, Israel; Cinvestav-IPN, Mexico

## Abstract

The response to transforming growth factor-β (TGF-β) depends on cellular context. This context is changed in mitosis through selective inhibition of vesicle trafficking, reduction in cell volume and the activation of mitotic kinases. We hypothesized that these alterations in cell context may induce a differential regulation of Smads and TGF-β receptors. We tested this hypothesis in mesenchymal-like ovarian cancer cells, arrested (or not) in mitosis with 2-methoxyestradiol (2ME2). In mitosis, without TGF-β stimulation, Smad3 was phosphorylated at the C-terminus and linker regions and localized to the mitotic spindle. Phosphorylated Smad3 interacted with the negative regulators of Smad signaling, Smurf2 and Ski, and failed to induce a transcriptional response. Moreover, in cells arrested in mitosis, Smad3 levels were progressively reduced. These phosphorylations and reduction in the levels of Smad3 depended on ERK activation and Mps1 kinase activity, and were abrogated by increasing the volume of cells arrested in mitosis with hypotonic medium. Furthermore, an Mps1-dependent phosphorylation of GFP-Smad3 was also observed upon its over-expression in interphase cells, suggesting a mechanism of negative regulation which counters increases in Smad3 concentration. Arrest in mitosis also induced a block in the clathrin-mediated endocytosis of the type II TGF-β receptor (TβRII). Moreover, following the stimulation of mitotic cells with TGF-β, the proteasome-mediated attenuation of TGF-β receptor activity, the degradation and clearance of TβRII from the plasma membrane, and the clearance of the TGF-β ligand from the medium were compromised, and the C-terminus phosphorylation of Smad3 was prolonged. We propose that the reduction in Smad3 levels, its linker phosphorylation, and its association with negative regulators (observed in mitosis prior to ligand stimulation) represent a signal attenuating mechanism. This mechanism is balanced by the retention of active TGF-β receptors at the plasma membrane. Together, both mechanisms allow for a regulated cellular response to TGF-β stimuli in mitosis.

## Introduction

The canonical signaling event induced by transforming growth factor-β (TGF-β) ligands initiates with the ligand-mediated enhancement of the hetero-oligomerization of the type I and type II serine-threonine kinase TGF-β receptors (TβRI and TβRII) at the plasma membrane (reviewed in [1]). This is followed by the trans-activation of TβRI by TβRII, the TβRI-induced phosphorylation of Smad2/3 on the C-terminal SSXS motif, the hetero-oligomerization of phosphorylated Smad2/3 with Smad4 and the nuclear translocation of this hetero-complex; resulting in the Smad-mediated transcriptional activation/repression of a broad repertoire of target genes [2]. In addition to their phosphorylation by TβRI, Smads2/3 are regulated through multiple mechanisms, including de-phosphorylation, nuclear export, degradation, kinesin-mediated transport and phosphorylation on residues other than the C-terminal SSXS motif [3], [4], [5], [6]. Phosphorylation of the inter-domain linker region of receptor-activated Smad proteins is involved in the regulation of Smad activity and turnover through the mediation of interactions with different cellular factors, such as ubiquitin ligases [6], [7], [8], [9], [10]. Ubiquitin ligases negatively regulate Smad activity by directing it towards degradation [6], [7], [9], [11], or by a recently identified multiple mono-ubiquitination mechanism [12]. Importantly, different phosphatases may mediate the de-phosphorylation of the C-terminus and linker regions of receptor activated Smads [13]. Smad activity is also negatively regulated by Ski and SnoN [14]. Of note, binding of Ski and SnoN to Smad3 has recently been reported to be enhanced in mitosis [15].

In spite of a high degree of structural similarity, Smad2 and Smad3 may be under differential regulation and perform unique functions. Thus, Smad2 and Smad3 differ in their ability to directly bind DNA [16], in their potential to induce the acquisition of metastatic attributes in ovarian and breast cancer cells [17], in their functional dependence on regulatory elements of microtubule mediated transport [18], [19], and in their regulation by the cell cycle [20].

In addition to the regulation at the Smad level, the termination of the TGF-β signal is also regulated at the level of the receptors. In this context, the activity of the TGF-β receptors may be altered through: the phosphorylation of multiple residues [21], intracellular trafficking and/or localization to membrane microdomains [22], [23], [24], [25] and proteasome-mediated degradation [26], [27], [28]. Of note, the mechanisms which mediate the termination of the TGF-β signal appear to depend on cell type and context [29].

In the mitotic cell, the structure of the cytoskeleton is altered, endocytosis is selectively inhibited, endosomal recycling is arrested and the nuclear membrane is disassembled [30], [31], [32], [33], [34]. Mitotic progression also involves the timely activation/de-activation of a broad repertoire of kinases with hundreds of different molecular targets [35]. Smads are phosphorylated by cyclin dependent kinases (Cdks, [36]), the mono-polar spindle kinase 1 (Mps1, [37]) and extra-cellular signal regulated kinase (ERK) [38], all of which are active at different stages of the cell cycle. Due to the demonstrated potential of these factors to regulate the TGF-β signal, the altered cellular environment of the mitotic cell is expected to modify the TGF-β signal output.

ES-2 and HEY ovarian cancer cells harbor mutations in the B-Raf oncogene [39] and performed aggressively in an intra-peritoneal xenograft model [40]; in accord with their classification as an advanced stage type I ovarian carcinoma model, a malignancy characterized by a stepwise progression from precursor lesions to aggressive tumors [41], that is also typically refractory to a number of first-line chemotherapy agents.

2-Methoxyestradiol (2ME2), a metabolite of 17β-estradiol, has demonstrated anti-angiogenic, anti-proliferative and pro-apoptotic activities. At clinically relevant doses, 2ME2 impairs microtubule dynamics and function, without causing gross depolymerization of the microtubule network. As a consequence, 2ME2 perturbs the correct assembly and function of the mitotic spindle, activates the spindle assembly checkpoint and causes metaphase arrest [42].

Here, we show that in mesenchymal-like ovarian cancer cells, 2ME2-mediated arrest in mitosis induced the phosphorylation of Smad3 and a reduction in Smad3 levels, prior to TGF-β addition. Moreover, in cells arrested in mitosis with 2ME2, the proteasome-mediated termination of the TGF-β signal is hampered, the endocytosis of TβRII is inhibited and the levels of C-terminus phosphorylated Smad3 are sustained at late time points after ligand addition.

## Materials and Methods

### Cells and Plasmids

HEY, Ovcar3, Skov3 and Caov3 human ovarian cancer cell lines were obtained from the ATCC and were a kind present of Prof. Shimon Slavin (Sourasky Medical Center, Tel Aviv). ES-2 human ovarian cancer cells (from the ATCC collection) were a kind gift of Dr. Michal Neeman (Weizmann Institute, Rehovot, Israel). With the exception of Ovcar3 cells, all cells were grown in (DMEM, 10% Fetal Calf Serum (FCS), Penicillin (25 µg/ml), Streptomycin (40 µg/ml), and Glutamine (5 mM); all from Biological Industries, Beit HaEmek, Israel). Ovcar3 cells were grown in (RPMI, 20% Fetal Calf Serum (FCS), Penicillin (25 µg/ml), Streptomycin (40 µg/ml), Glutamine (5 mM) and Sodium Pyruvate (1 mM)); all from Biological Industries, Beit HaEmek, Israel. Myc-TβRII-GFP was generated by inserting the myc-TβRII sequence [43] into the pEGFP-N1 vector (Clontech). The entire sequence was confirmed before use. The ES-2 cell line, stably expressing myc-TβRII-GFP was generated by transfection (Lipofectamine, Invitrogen) of myc-TβRII-GFP and selection with G418 (Calbiochem, 1.5 mg/ml). The TGF-β responsiveness of this cell line was confirmed through their stimulation with TGF-β1 (5 ng/ml, 1 h) and the assessment of the C-terminus Smad3 phosphorylation (data not shown). The plasmids encoding for myc-TβRII, GFP-Smad3 and the (CAGA)_12_-Luc reporter construct were all kind gifts of Prof. Yoav Henis (Tel Aviv University, Tel Aviv, Israel).

### Drugs and Treatments

Reagents were employed at the following final concentrations and treatment periods: 2-Methoxyestradiol (2ME2), 4.4 µM, 16 h; β-cyclodextrin, 5 mM; cycloheximide, 300 µM; dorsomorphin, 4 µM; nocodazole, 50 µM; SB431542, 10 µM; U0126, 230 µM; proteasome inhibitors: Acetyl-L-Leucyl-L-Leucyl-L-Norleucinal (ALLN), 25 µM and MG132 20 µg/ml; all from Sigma Aldrich; A83-01, 1 µM (Tocris); reversine, 5 µM (Cayman). In control treatments, a similar concentration of vehicle (DMSO or ethanol) was employed. TGF-β1 was from PeproTech Inc, and was employed at 5 ng/ml. Ligand incubations were in 0.5% FCS (with or without additional treatments), after 1 h starvation in the same medium. Hypotonic medium was (KCl, 75 mM; glucose, 4 gr/l; FCS, 1%; Hepes, 20 mM, pH = 7.4; CaCl_2,_ 0.25 mM; MgCl_2_, 1 mM; Glutamine, 5 mM; non essential amino acids). Hypertonic medium was DMEM-based medium supplemented with 0.45 M sucrose.

### Immunochemicals

The following antibodies and reagents were employed in the present study (all used at: WB: 1∶1000, IF: 1∶100, unless specified otherwise): α-e-cadherin, α-pSmad3C (raised against phospho-serines 423/425 of Smad3 and cross-reactive with C-terminus phosphorylated Smad2), α-tSmad3, all from Cell Signaling; α-phospho-Smad3(179) (threonine 179; WB: 1∶500; Assay Biotech); α-Mps1 (IF: 1∶333; Abcam); α-tubulin-α (Bio-legend); α-clathrin heavy chain (Novus); α-vimentin (IF: 1∶50; Sigma Aldrich); α-Smad2/3 (WB: 1∶500), α-Smurf2 (WB:1500), α-Ski, all from Santa Cruz. Rhodamine-conjugated phalloidin (1∶600; Molecular Probes). The H23 antibody, against the tandem repeat of MUC1 was prepared by Prof. I Keydar (Tel Aviv University; IF: 20 µg/ml). The α-myc tag hybridoma (9E10) was a generous gift of Prof. Yoav Henis (Tel Aviv University), the antibody was labeled with Alexa-546 (Molecular Probes labeling kit, according to manufacturer’s instruction). Secondary Alexa 488-, 555- and 647-conjugated antibodies (IF: 1∶200; Molecular Probes), and HRP-conjugated (WB: 1∶12,500; Jackson).

### Cell Lysis and Immunoblotting

An equal number of cells were lysed in (NaCl, 150 mM; Hepes, 10 mM pH 7.4; Igepal CA-630, 0.5%; Triton, 1%; protease and phosphatase inhibitors (Sigma)). Cell-lysates were immunoblotted as described before [34]. All immunoblots that appear in the present manuscript are representative of 3–4 independent experiments, unless indicated otherwise.

### Immunoprecipitation

10^8^ cells were lysed in IP lysis buffer [420 mM NaCl, 50 mM Hepes (pH 7.8), 5 mM EDTA, 1% NP-40 and 3% DTT], and subjected to immunoprecipitation with α-pSmad3C (over night, 4°C) followed by incubation with protein A-sepharose beads (GE Healthcare; 2 h, 4°C). After extensive washes and elution, immunoprecipitates were separated by SDS-PAGE and immunoblotted as indicated above.

### Immunofluorescence

Cells were plated onto glass coverslips in 24-well plate (5*10^4^ cells/well) and treated with indicated substance ∼16 h post plating. Coverslips were washed twice with cold PBS (4°C), fixed (4% paraformaldehyde, 20 min, room temperature), blocked and permeabilized (3% BSA; 0.1% triton, in PBS; 1 h), stained with primary antibody (1% BSA; 0.1% triton, in PBS; 1 h; staining solution) and secondary antibody (1∶200 dilution in staining solution supplemented with DAPI stain, 30 min). Mounting was with Fluorescence Mounting medium (Golden Bridge).

### Imaging, Acquisition, Processing and Quantitation

Cells were prepared for imaging as in [34] and imaged with a spinning disk confocal microscope setup: Zeiss 100x, NA 1.4; Yokogawa CSU-22; Zeiss fully–automated -inverted 200 M; solid state lasers (473, 561 and 660 nm); piezo controlled Z-stage all under the command of Slidebook™. Images employed for co-localization calculations were acquired with an HQ2 CCD camera (Photometrics). Typically, a 1X1 binning was employed, yielding a pixel size of 0.065 microns. The micrograph inset images were processed with the NearestNeighbors deconvolution algorithm of Slidebook™ for clarity. Alternatively, images were acquired with an Evolve EMCCD camera (Photometrics; 100×lens, 1×1 binning, yielding a pixel size of 0.16 microns).

### q-RT-PCR

RNA was isolated using the EZ-RNA kit (Biological Industries, Beit HaEmek, Israel). RT-PCR was carried out with 2 µg RNA, employing M-MLV enzyme reverse transcriptase (PROMEGA) according to the manufacturer’s instructions. Real-time PCR was carried out with the Rotor Gene 6000 system (Corbett, Australia), employing Absolute Blue SYBER Green ROX (Thermo scientific). Non-template controls (NTC) and quantitative standards (GapDH) were included. Analysis was with the Rotor Gene 6000 system series software. Primers were: Smad7- F: 5′-CGAACTAGAGTCTCCCCCCC-3′, R: 5′-GAATCTGAAAGCCCCCCAG-3′. PAI-1- F: 5′- CAACCCCACAGGAACAGTCC-3′, R: 5′- TTTGTCCCAGATGAAGGCGT-3′. SnoN- F: 5′-GAATATGCAGGACAGTTGGCAG-3′, R: 5′-GCTTCCCGTTCCTGTCTGATG-3′. fibronectin- F: 5′-CAAAGCAAGCCCGGTTGT-3′, R: 5′-AACCAACGCATTGCCTAGGTAG-3′. Smad3- F: 5′- TCGAGCCCCAGAGTAATATT-3′, R: 5′-AGAACCTGCGTCCATCGTG-3′. Importantly, in assays in which the transcriptional response of cells arrested in mitosis was addressed, we employed a cell detachment procedure to enrich the proportion of mitotic cells in this defined experimental condition. Specifically, sub-confluent ES-2 cell cultures (grown in 25 mm cell culture flasks, and treated with 2ME2 or vehicle) were gently frapped (a total of 40 times and gentle vortexing) before the collection of the medium containing detached cells (including the medium of 2 washes with PBS). Cells which detached upon this treatment were markedly enriched in terms of their 4 n DNA content, as measured by FACS (data not shown).

### Cell Proliferation

5×10^3^ cells/well were plated overnight in 96 well plates, and incubated as specified in the figure legend. Fresh growth medium was replaced every 24 h. Cells were fixed (4% formaldehyde), stained (0.5% methylene blue/0.1 M sodium borate pH 8.5), and dissolved in 0.1 M HCl. Absorbance was measured at 595 nm (6 repetitions/time-point/condition).

### Transcriptional Activation Assay

Cells were cultured in 96-well plates and transfected with 0.16 µg/well of (CAGA)_12_-Luc and 0.05 µg/well pRL-TK (Renilla luciferase, Promega). At 16–24 h post transfection, cells were treated or not with 2ME2 (4.4 µM, 16 h), starved (0.5% FCS, 1 h) in medium containing 2ME2 or vehicle, and stimulated or not with 5 ng/ml TGF-β1 (6 h). Cells were then lysed and analyzed with the DLR assay system. The results were normalized for transfection efficiency using the Renilla luminescence.

### myc-TβRII-GFP Endocytosis Experiment

ES-2 cells, stably expressing myc-TβRII-GFP, were plated onto glass coverslips (16 h) and pre-treated or not with 2ME2 (4.4 µM, 16 h) or hypertonic medium (medium supplemented with 0.45 M sucrose; 30 min) or transfected with siRNA directed against the clathrin heavy chain (48 h). Cells were fed Alexa-546-labeled α-myc antibodies (20 µg/ml, 30 min, 37°C; in the same medium as pre-treatments). Cells were subsequently cooled to 4°C and the membrane bound sub-population of Alexa-546-α-myc was labeled with goat-anti-mouse Alexa-647 antibodies. Entire cell volumes were acquired by confocal microscopy. Specific signals were identified by intensity based segmentation (Slidebook™) and the % of Alexa-546 signal which did not overlap with Alexa-647 signal was interpreted as internalized/sequestered receptor.

### Measurement of the Turnover Myc-TβRII-GFP

The turnover of myc-TβRII-GFP was measured through immunoblot-based measurements of total myc-TβRII-GFP cellular content and through an immunofluorescence-based measurement of the levels of myc-TβRII-GFP exposed at the plasma membrane. For immunoblots, sub-confluent cultures of ES-2 cells stably expressing myc-TβRII-GFP, arrested in mitosis (or not) with 2ME2, were pre-incubated (20 min) with serum depleted medium containing cycloheximide and supplemented (or not) with proteasome inhibitors (ALLN + MG132). Subsequently, cells were either transferred to 4°C or incubated for 3 h in the same medium supplemented with TGF-β1 (5 ng/ml). Cells were subsequently analyzed by immunoblotting with α-myc and α-GFP (not shown) antibodies. Immunofluorescence-based experiments were essentially similar with the following differences: cells were cultured on glass coverslips, detection of myc-TβRII-GFP localized at the plasma membrane was by α-myc immunostaining of live intact cells at 4°C, detection was by confocal microscopy. Specifically, fields of cells (containing ∼350 cells) were imaged with 405 nm (for DAPI staining) and 561 nm illumination (for Alexa-546-GαM). Total fluorescence signals obtained in these conditions were employed for the calculation of the normalized Alexa-546-GαM signal (Alexa-546-GαM/DAPI). 3 independent experiments were performed, yielding 20 fields for each experimental condition.

### siRNA-mediated Expression Knockdown and Transferrin Uptake Experiments

Transfection of siRNA was performed with INTERFERin™ (Polypus) according to manufacture’s instructions. The following siRNA oligos (Dharmacon) were employed: α-adaptin - (GAGCAUGUGCACGCUGGCCAGCU); clathrin heavy chain- (GCAATGAGCTGTTTGAAGA), Smurf2– (a combination of: GCAAAAGUAUCCCUGUUAA; CCACUUUGUUGGACGAAUA; AGAAUACGCUUGAUCCAAA; GUUAAUGACUGGAAGGUAA) and a commercially supplied scrambled sequence (negative control). Transfections were carried out with cells grown on 12 mm coverslips (in 24-well plates) for microscopy-based experiments, and in 35 mm dishes for experiments based on immunoblotting. Cells were assayed at 48 h post transfection. For experiments involving transferrin uptake, siRNA transfected cells were starved for 1 h, activated with TGF-β (5 ng/ml, 1 h) and incubated with fluorescently-labeled transferrin (100 µg/ml, 10 min; Molecular Probes) diluted in the TGF-β containing medium. Cells were subsequently fixed, permeabilized, stained for Smad3 and imaged by confocal microscopy.

### Cell Cycle Analysis by Flow Cytometry

Cells were harvested, washed twice with phosphate-buffered saline, and resuspended in 0.5 ml of phosphate-buffered saline containing 0.1% Triton X-100 and 50 µg/ml propidium iodide. Samples were analyzed by fluorescence-activated cell sorter (FACS) flow cytometry (FACSort, BD Bioscience) using CellQuest Pro™ software.

### Medium-transfer Assay

“Donor cultures” were grown to semi-confluence in 60 mm plates, treated with 2ME2 or vehicle (16 h, 4.4 µM) and serum starved (1.5 ml, 0.5% FCS with the respective treatments, 1 h) prior to stimulation with TGF-β1 (1–4 h, 5 ng/ml, in treatment medium). Medium from these “donor cultures” was collected and transferred to pre-starved naïve “reporter cultures” (750 µl medium per 6-well plate, in duplicates) for 1 h of stimulation.

## Results

### Mesenchymal-like Ovarian Cancer Cells are TGF-β Responsive

**Figure 1:**
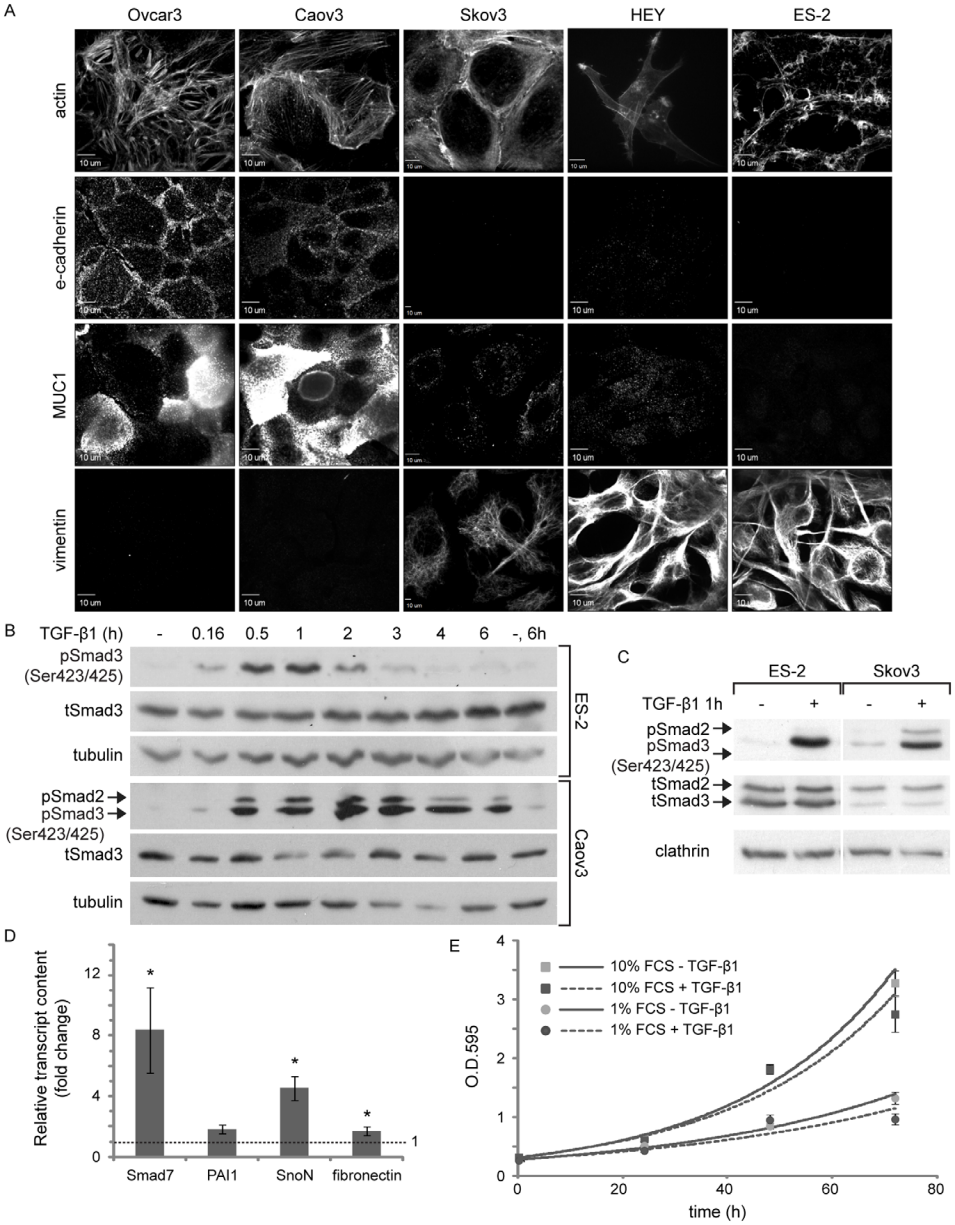
ES-2 ovarian cancer cells have a mesenchymal phenotype and respond to TGF-β1. A, Panels depict confocal micrographs of different ovarian cancer cell lines (as specified) immuno-stained against e-cadherin, MUC1 and vimentin; or labeled for their actin content using rhodamine-conjugated phalloidin. Note the difference in the pattern of expression of phenotypic markers amongst epithelial-like cells (Ovcar3 and Caov3) and mesenchymal-like cells (ES-2 and HEY). Bar, 10 µm. B, ES-2 and Caov3 cells were stimulated with TGF-β1 (5 ng/ml, here and throughout the manuscript) or vehicle for the indicated times and immunoblotted with α-pSmad3C, α-tSmad3 and α-tubulin (employed here and throughout the manuscript as a protein loading control) antibodies. C, ES-2 and Skov3 cells were stimulated with TGF-β1 or vehicle and immunoblotted with α-pSmad3C, α-tSmad2/3 and α-clathrin (employed here and throughout the manuscript as a protein loading control) antibodies. D, qRT-PCR of Smad7, PAI-1, SnoN and Fibronectin. Bar graph depicts the average ± SEM fold change in normalized transcript content in TGF-β1-treated cultures (2 h) as compared to untreated. Smad7∶ 8.4±2.82, n = 8; p<0.01; PAI1∶ 1.82±0.27, n = 6; p<0.06; SnoN: 4.54±0.77, n = 8; p<0.01; fibronectin: 1.7±0.28, n = 9; p<0.03; all 1-tailed t-tests. E, Growth curve of ES-2 cells treated with TGF-β1 or vehicle. Cells were grown in medium supplemented with 1% or 10% FCS, with or without TGF-β1. Graph depicts the average ± SEM optical density at each time point of a typical experiment (O.D._595_, n = 6).

The objective of the present study was to characterize TGF-β signaling in mitosis in mesenchymal-like ovarian cancer cells. Initially, we characterized the profile of expression of phenotypic markers and the TGF-β responsiveness of our cellular models. ES-2 and HEY ovarian cancer cell lines exhibit activating mutations to the B-Raf oncogene and performed aggressively in an intra-peritoneal xenograft experimental model, supporting their classification as advanced-stage type I ovarian cancer cells [39], [40], [41]. These cells did not express the epithelial markers e-cadherin and mucin 1 (MUC1) while expressing vimentin, a typical marker of cells which have undergone epithelial-to-mesenchymal transition (Figure 1A). ES-2 and HEY cells also presented spindle-like morphology, concentrated polymerized actin at the leading edge (Figure 1A) and exhibited fast spreading kinetics on fibronectin (data not shown). These characteristics are in contrast to the expression pattern of phenotypic markers presented by the epithelial-like ovarian cancer cell lines Ovcar3 and Caov3 (high e-cadherin and MUC1 expression, no vimentin expression and a localization of actin to cell-cell junctions), and by the Skov3 cell line which presented a mixed pattern of marker expression (no e-cadherin expression, low MUC1 expression, considerable vimentin expression and a localization of actin to cell-cell junctions). From this characterization we conclude that ES-2 and HEY cells are of mesenchymal phenotype *in vitro*. Due to their similarity, the present study centers on ES-2 cells, while selected confirmatory experiments were performed with HEY cells (Figure S1).

**Figure 2:**
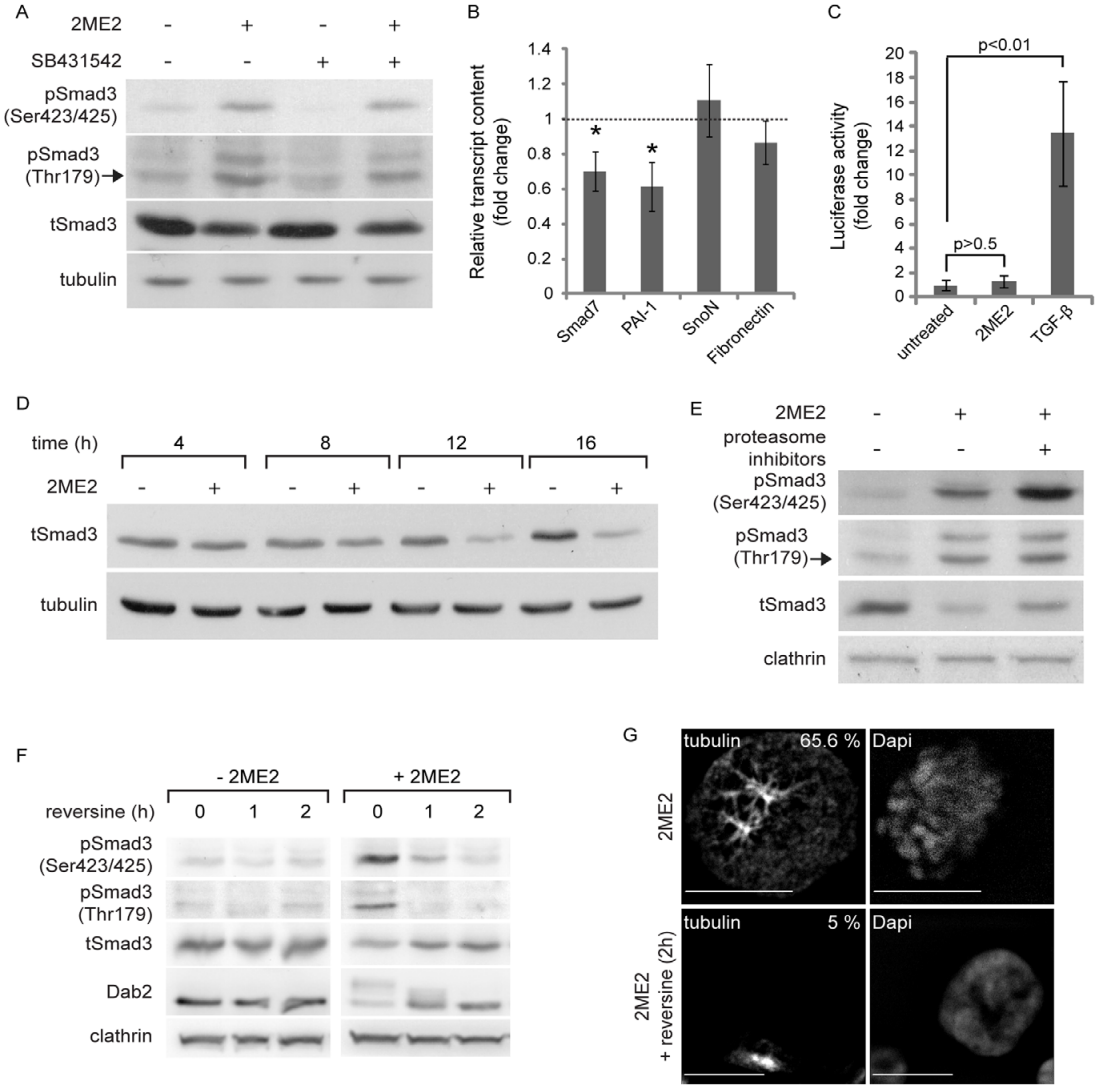
Mitotic arrest with 2ME2 induces Smad3 phosphorylation but not Smad-dependent transcription, and a reduction in Smad3 levels. A, α-pSmad3C, α-pSmad3(179), α-tSmad3 and α-tubulin immunoblot of ES-2 cells, treated with 2ME2, SB431542, their combination or vehicle. 2ME2-arrested cells show a 4.6±1.5 fold increase in Smad3 phosphorylated at the C-terminus (pSmad3C)/total-Smad3(tSmad3) ratio, n = 14; p<0.016, 1-tailed t-test; and 11.9±2.15 fold increase in Smad3 phosphorylated on Threonine 179 pSmad3(179)/tSmad3 ratio, n = 9; p<0.01, 1-tailed t-test. B, qRT-PCR of Smad7, PAI-1, SnoN and fibronectin. Bar graph depicts the average ± SEM fold change in normalized transcript content in 2ME2-treated cultures as compared to untreated. *, p<0.04, n = 8; all 2-tailed t-tests. C, Transcriptional activation assay. ES-2 cells, co-transfected with the (CAGA)_12_-Luc reporter construct and pRL-TK (see Material and Methods), were either arrested in mitosis with 2ME2 or stimulated with TGF-β1 (5 ng/ml, 6 h). TGF-β1 induced a 13.4±4.3 fold increase in the normalized luciferase signal, n = 3 independent experiments (6 wells/treatment/experiment); p<0.01, 1-tailed t-test. Arrest in mitosis with 2ME2 did not induce a significant change in luciferase signal; 1.33±0.44 fold, n = 3 independent experiments (6 wells/treatment/experiment); p>0.6, 2-tailed t-test. D, α-tSmad3 and α-tubulin immunoblots of ES-2 cells, treated with 2ME2 or vehicle for the indicated times. At 16 h of 2ME2-arrest the tSmad3/tubulin ratio was reduced to 0.37±0.05 fold of the initial amount, n = 10; p<0.01, 1-tailed t-test. E, α-pSmad3C, α-pSmad3(179), α-tSmad3 and α-clathrin immunoblot of ES-2 cells, arrested in mitosis with 2ME2, and treated or not with proteasome inhibitors (MG132 and ALLN, 3 h). Upon proteasome inhibition, the (pSmad3C/tSmad3)/clathrin ratio increased by 2.3±0.2 fold, n = 5; p<0.02, 1-tailed t-test. F, α-pSmad3C, α-pSmad3(179), α-tSmad3, α-Dab2 and α-clathrin immunoblots of ES-2 cells, treated with 2ME2 or vehicle followed by addition of reversine (5 µM, 0–2 h). G, Confocal micrographs of tubulin and DNA of 2ME2-arrested ES-2 cells, treated or not with reversine (2 h). Bar, 10 µm. Numbers in the upper right-hand corner of the micrographs are the percentage of cells presenting a rounded morphology and a spindle-like organization of the microtubules.

Continuous incubation of ES-2 cells with TGF-β1 revealed a single phosphorylated Smad3 band and a bell-shaped profile of Smad3 activation, with a prominent drop in C-terminally phosphorylated Smad3 (pSmad3C) levels occurring already after 2 hours of ligand addition (Figure 1B). A similar pSmad3C staining pattern and activation/de-activation profile was observed with HEY cells (Figure S1A). In contrast, continuous incubation of Caov3 cells with TGF-β1 induced a prolonged activation of Smad3, with considerable pSmad3C levels at 6 h after ligand addition (Figure 1B). An identical prolonged profile of pSmad3C levels was observed upon the activation of Ovcar3 cells (data not shown). Moreover, immunoblotting with antibodies directed against the C-terminal phosphorylated residues of Smad3 revealed a doublet staining pattern in Caov3, Ovcar3 and Skov3 cells (Figure 1B–C and data not shown). The higher molecular weight band of the doublet, which was absent in ES-2 and HEY cells, overlapped with anti-Smad2 staining (see Skov3 cells, Figure 1C). These data suggest that Smad2 activation may be less pronounced in mesenchymal-like ovarian cancer cells than in their epithelial-like counterparts. Of note, in ES-2 cells, no phosphorylation of threonine 179 (denominated pSmad3(179)), which localizes to the inter-domain linker region of Smad3, was observed either prior to or following TGF-β1 addition (data not shown). Importantly, in ES-2 and HEY cells TGF-β1 induced a transcriptional response seen by the ligand-mediated increases in the transcripts of TGF-β target genes (Smad7, plasminogen activator inhibitor-1 (PAI-1), SnoN and fibronectin; Figure 1D and Figure S1C) and by the transcriptional activation of the (CAGA)_12_-Luc reporter construct (Figure 2C). However, TGF-β1 did not induce a pronounced growth inhibitory response in either mesenchymal-like cell type (shown here ES-2 cells; Figure 1E). Taken together, these experiments establish ES-2 cells as TGF-β-responsive type I ovarian cancer cells of mesenchymal-like phenotype.

### Ligand-independent Phosphorylation of Smad3 in Mitosis

Arrest in mitosis with nocodazole induces the receptor-independent phosphorylation of Smads 2 and 3 in a variety of epithelial cell types (Hela, Mv1Lu and SW480M4 cells) and an increase in the transcription of the (CAGA)_12_-Luc reporter construct [37]. Here, we employed 2-methoxyestradiol (2ME2) which arrests cells at the spindle assembly checkpoint without causing gross microtubule depolymerization [42]. Arrest in mitosis with 2ME2 induced a significant phosphorylation of Smad3 at its C-terminus (∼5 fold increase in pSmad3C/tSmad3 ratio; Figure 2A) and on threonine 179 (∼12 fold increase in the pSmad3(179)/tSmad3 ratio; Figure 2A). These phosphorylations of Smad3 in mitosis were unaffected by the addition of the TGF-β receptor kinase inhibitor SB431542 (Figure 2A), suggesting a lack of involvement of the TGF-β receptor kinase, and in accord with [37]. Similarly, dorsomorphin and A83-01 (inhibitors of the kinase activity of receptors of the TGF-β superfamily of ligands) were devoid of effects on the mitosis-induced phosphorylations of Smad3 (Figure S2A–B). Notably, arrest in mitosis also induced an increase and change in pattern of the C-terminus phosphorylation of Smad1/5/8. However, this phosphorylation was sensitive to dorsomorphin, suggesting the involvement of the Bone Morphogenetic Protein receptors in this process (Figure S2A). Importantly, the receptor-independent phosphorylation of Smad3 did not induce a transcriptional response of endogenous TGF-β target genes (Figure 2B), or an enhancement of luciferase activity of the (CAGA)_12_-Luc reporter construct (Figure 2C, second column). This is in sharp contrast to the ligand-induced activation of these same target genes (Figure 1D) and to the ligand-induced increase in (CAGA)_12_-Luc activity (Figure 2C, third column) in cycling cells. Concomitant to the phosphorylation of Smad3 at its C-terminus and threonine 179, 2ME2 also induced a progressive reduction in Smad3 protein levels (culminating at 16 h treatment, ∼0.35 fold of the initial tSmad3/tubulin ratio; Figure 2D). Arrest in mitosis of HEY cells resulted in a similar reduction of tSmad3 levels (Figure S1B). To probe for the involvement of proteasome-mediated degradation in the observed reduction in tSmad3 levels, we treated cells arrested in mitosis with a combination of proteasome inhibitors (see methods). Proteasome inhibition (3 h) resulted in a marked accumulation of pSmad3C (∼2.5 fold), a lesser increase in tSmad3 (which did not reach statistical significance), and no increase in pSmad3(179) (Figure 2E). These results are in accord with the notion that in mitosis pSmad3C is a small fraction of tSmad3 and is targeted by the proteasome. The lack of accumulation of pSmad3(179) suggests that either this phosphorylation does not coincide with the C-terminus phosphorylation of Smad3 on the same subset of molecules, or that pSmad3(179) levels may be regulated by additional means such as site-specific phosphatases. Of note, arrest in mitosis with 2ME2 induced only a slight reduction in Smad3 mRNA, which failed to reach statistical significance (measured by qRT-PCR, 0.8±0.21 fold of untreated, n = 6; p>0.2, 1-tailed t-test), suggesting a minimal contribution of a reduction in transcription to the observed decrease of tSmad3 levels. However, additional mechanisms such as a differential regulation of protein synthesis may also contribute to the reduction of tSmad3 levels observed in mitosis. Taken together, these data suggest a connection between the receptor-independent phosphorylation of Smad3 in mitosis and the reduction in its levels. The mono-polar-spindle kinase 1 (Mps1) was recently shown to phosphorylate Smads in mitosis [37]. Reversine is a specific inhibitor of Mps1 [44]. Treatment with reversine of cells arrested in mitosis induced a marked decrease in pSmad3C and pSmad3(179) levels, while causing a concomitant increase in tSmad3 levels (Figure 2F). However, Mps1 activity is necessary for the maintenance of the spindle assembly checkpoint [44]. Indeed, a 2 h treatment of 2ME2-arrested cells with reversine reduced the percentage of cells presenting mitotic features (rounded morphology, condensed chromosomes, and a spindle-like organization of microtubules, Figure 2G) and the phosphorylation of Dab2 (a mitotic phosphoprotein, [34]; Figure 2F). These data support the involvement of Mps1 activity on the here-reported Smad3-related phenomena, but fall short of differentiating between a direct phosphorylation of Smad3 by Mps1, from the function of the latter as a regulator of mitosis.

The linker region of Smad3 was reported to be phosphorylated by cyclin dependent kinases (cdks, [36]) and by ERK [38]. Inhibition of cdks with roscovitine reduced the phosphorylation of Smad3 and the decrease in its protein levels, but impaired the ability of 2ME2 to induce an arrest in mitosis (data not shown, [34]). Arrest of ES-2 cells in mitosis with 2ME2 induced a marked activation of ERKs 1 and 2, which was fully inhibited by U0126 (Figure S3A). In addition, U0126 reduced both the C-terminus and the threonine 179 phosphorylation of Smad3 induced by 2ME2 (Figure S3A) and induced a parallel increase in tSmad3 levels. However, these effects on Smad3 occurred in the context of a reduction in the percentage of cells arrested in mitosis (Figure S3B); preventing once again the dissection of the direct ERK-mediated effects on Smad3 and its potential functions in regulating mitosis. Taken together, these data firmly establish a connection between the arrest in mitosis of ES-2 cells and the Smad3-related phenomena (phosphorylation and reduction in tSmad3 levels), and support the notion of regulatory roles for Mps1, cdks and MEK/ERK in these processes. However, these data cannot exclude a putative contribution to these processes of an altered regulation of phosphatases in cells arrested in mitosis.

**Figure 3:**
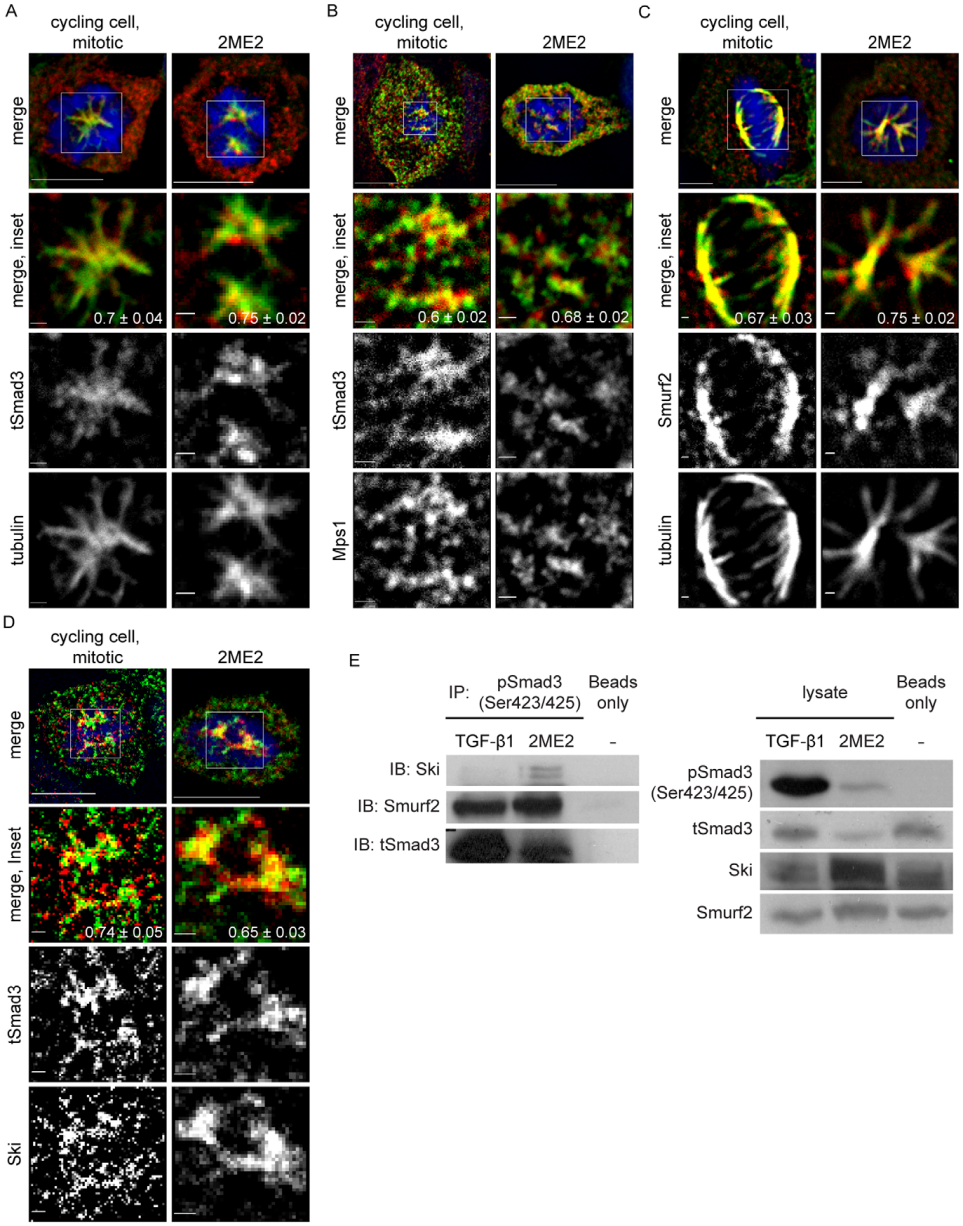
Localization of Smad3, Smurf2, Ski and Mps1 to the mitotic spindle and co-immunoprecipitation of phosphorylated Smad3, Ski and Smurf2 in 2ME2-arrested cells. A–D, Panels depict a single confocal mid-plane of imaged cells. Bar, 10 µm in upper panel and 1 µm in inset images. The Pearson’s Correlation Coefficient (PCC) of images acquired with 473 nm and 561 nm illumination was calculated with the JaCop plugin of the ImageJ™ software (presented as PCC ± SEM). A, Confocal micrographs of tSmad3, α-tubulin and DNA, of unperturbed-mitotic or 2ME2-arrested ES-2 cells. B, Confocal micrographs of tSmad3, Mps1 and DNA of unperturbed-mitotic or 2ME2-arrested ES-2 cells. C, Confocal micrographs of Smurf2, α-tubulin and DNA of unperturbed-mitotic or 2ME2-arrested ES-2 cells. D, Confocal micrographs of tSmad3, Ski and DNA of unperturbed-mitotic or 2ME2-arrested ES-2 cells. E, Immunoprecipitation with α-pSmad3C or sepharose beads alone (control) of lysates of cells treated with TGF-β1 (1 h) or 2ME2 (16 h). Left panel: Immunoprecipitates were separated by SDS-PAGE and immunoblotted with α-Ski, α-Smurf2 and α-tSmad2/3 antibodies. In 2ME2-arrested cells, Ski and Smurf2 co-immunoprecipitated with pSmad3C at 7.7±1.7 fold and 2±0.25 fold (respectively) higher levels than in TGF-β- stimulated cells, (n = 3; p<0.02, 1-tailed t-test). Right panel: 5% of the lysates, prior to immunoprecipitation, were immunoblotted with α-pSmad3C, α-tSmad3, α-Ski and α-Smurf2 antibodies.

Mps1, Smads, the ubiquitin ligase Smurf2 and the Smad inhibitor Ski were reported to localize to the mitotic spindle in different cell types [44], [45], [46], [47]. A confocal analysis of ES-2 cells, either undergoing unperturbed mitosis or arrested in mitosis with 2ME2, and stained for these factors and α-tubulin, revealed their co-localization at the mitotic spindle (Figure 3A–D). The notion of their co-localization is supported by the Pearson’s correlation coefficient values of the distribution patterns of Smad3 and tubulin, Smurf2 and tubulin, Ski and tubulin and Mps1 and Smad3 (Figure 3 A–D, lower right-hand corner of merged insets). The lack of transcriptional activation following the receptor-independent C-terminus phosphorylation of Smad3, the concomitant phosphorylation of the Smad3 C-terminus and threonine 179 and the accumulation of pSmad3C upon inhibition of proteasomal degradation, suggested a differential and negative regulation of pSmad3C in mitosis. To substantiate this notion, we carried out an immunoprecipitation assay aimed at comparing the degree of association of Smurf2 and Ski with pSmad3C in cells arrested in mitosis and in cycling cells activated with TGF-β1 (Figure 3E). Calculation of the ratios of Ski/tSmad3 and Smurf2/tSmad3 in the anti-pSmad3C immunoprecipitates in the two conditions, revealed a 7.7±1.7 fold and 2±0.25 fold increase (respectively) of these ratios in the cells arrested in mitosis relative to the TGF-β1-activated cells. These data are in line with the negative regulation of pSmad3C in mitosis. Probing of the immunoprecipitates with anti-pSmad3(179) antibodies yielded inconsistent results, with pSmad3(179) being sometimes weakly detected in the 2ME2-treated sample (data not shown). To directly probe for an involvement of Smurf2 in the 2ME2-induced reduction in tSmad3 levels, we reduced the Smurf2 content of ES-2 cells with siRNA, arrested cells in mitosis and probed for tSmad3 and pSmad3C by immunoblotting. A ∼70% reduction in Smurf2 induced only minor alterations in the reduction of tSmad3 (Figure S4). These data are in line with the possible involvement of additional ubiquitin ligases in the observed reduction of tSmad3 levels and/or with the proposed regulation of Smad3 by Smurf2 through multiple-mono-ubiquitination, which may inhibit Smad activity without inducing its degradation [12].

**Figure 4:**
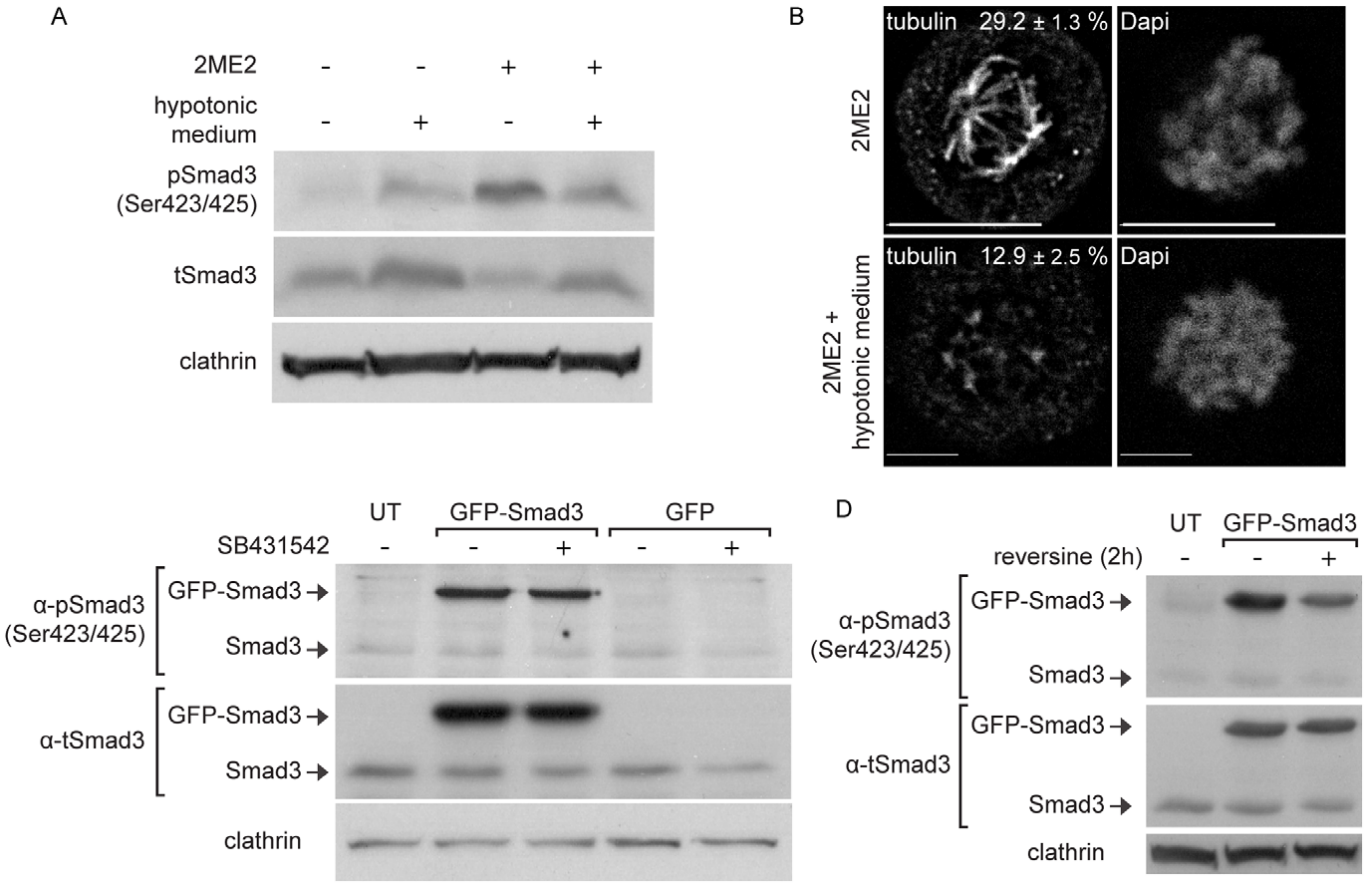
Receptor-independent phosphorylation of Smad3 is sensitive to an increase in the volume of mitotic cells and occurs upon GFP-Smad3 over-expression. A, α-pSmad3C, α-tSmad3 and α-clathrin immunoblot of ES-2 cells treated with 2ME2 or vehicle, hypotonic medium (2 h; as described in Materials and Methods) or their combination. 2ME2-arrested cells treated with hypotonic medium show 0.31±0.09 fold lower levels of the (pSmad3C/tSmad3)/clathrin ratio (n = 4; p<0.05, 1-tailed t-test), and 1.94±0.23 fold increase in tSmad3/clathrin ratio (n = 4; p<0.01, 1-tailed t-test), as compared to 2ME2-arrested cells grown in isotonic medium. B, Confocal micrographs of tubulin and DNA of 2ME2-arrested ES-2 cells, treated or not with hypotonic medium. Bar, 10 µm. Numbers represent the percentage of cellular tubulin that localizes to the spindle. In arrested cells treated with hypotonic medium, this percentage was 0.44±0.09 fold lower as compared to arrested cells grown in normal medium (n = 6; p<0.03, 1-tailed t-test). C, α-pSmad3C, α-tSmad3 and α-clathrin immunoblots of ES-2 cells, transfected with GFP-Smad3 or GFP constructs and treated with SB431542 (6 h) or vehicle. D, α-pSmad3C, α-tSmad3 and α-clathrin immunoblots of ES-2 cells, transfected with GFP-Smad3 and treated with reversine (5 µM, 2 h) or vehicle. Transfected cells treated with reversine showed 0.58±0.06 fold lower levels of (pGFP-Smad3C/tGFP-Smad3)/clathrin ratio (n = 4; p<0.05, 1-tailed t-test), as compared to untreated cells.

A prominent characteristic of mitotic cells in culture is their reduced volume in metaphase, which entails the condensation of their cytosol [30], [48], [49]. We hypothesized that this condensation of the cytosol may lead to an increase in the concentration of Smad3 and require a mechanism of negative regulation of Smad3 levels, in order to maintain a similar sensitivity to TGF-β stimulation in mitotic and cycling cells. To test this hypothesis, we initially probed if increasing the volume of cells arrested in mitosis affects the phosphorylation and reduction in Smad3 levels. To this end, we incubated ES-2 cells, arrested or not with 2ME2, with hypotonic medium and probed for pSmad3C and tSmad3 levels (Figure 4A). In arrested cells, hypotonic medium induced a significant decrease in pSmad3C levels and a parallel significant increase in tSmad3 levels. Moreover, a confocal microscopy analysis of the tubulin distribution of 2ME2-arrested cells under hypotonic treatment revealed a decrease in the fluorescent signal of microtubules in spindle-like structures, relative to cells in isotonic medium (Figure 4B). Thus, here too, a connection between Smad3 phosphorylation, the reduction of tSmad3 levels and the structure of the mitotic spindle can be established. In contrast, hypotonic medium treatment of cycling cells did not significantly alter the pSmad3C/tSmad3/clathrin ratio. To directly test if an increase in tSmad3 concentration entails its receptor-independent phosphorylation, we over-expressed GFP-Smad3 in ES-2 cells, treated them with either vehicle or SB431542 and followed Smad3 C-terminus phosphorylation by immunoblotting (Figure 4C). Over-expressed GFP-Smad3, phosphorylated at the SSXS motif, was readily detected upon immunoblotting. This phosphorylation was insensitive to SB431542 treatment, indicating a lack of involvement of the kinase activity of the TGF-β receptor. To examine the involvement of Mps1 in the phosphorylation of over-expressed GFP-Smad3, we treated transfected cells with reversine (Figure 4D). Here, a significant decrease in GFP-Smad3 C-terminal phosphorylation was observed. Of note, over-expression of GFP-Smad3 also induced the phosphorylation of threonine 179 (data not shown), suggesting that this phosphorylation site may also be an element of the negative regulation of Smad3 which is sensitive to increases in Smad3 levels.

### Reduced Proteasome-mediated Attenuation of the TGF-β Receptor-dependent Signal in Mitosis

**Figure 5:**
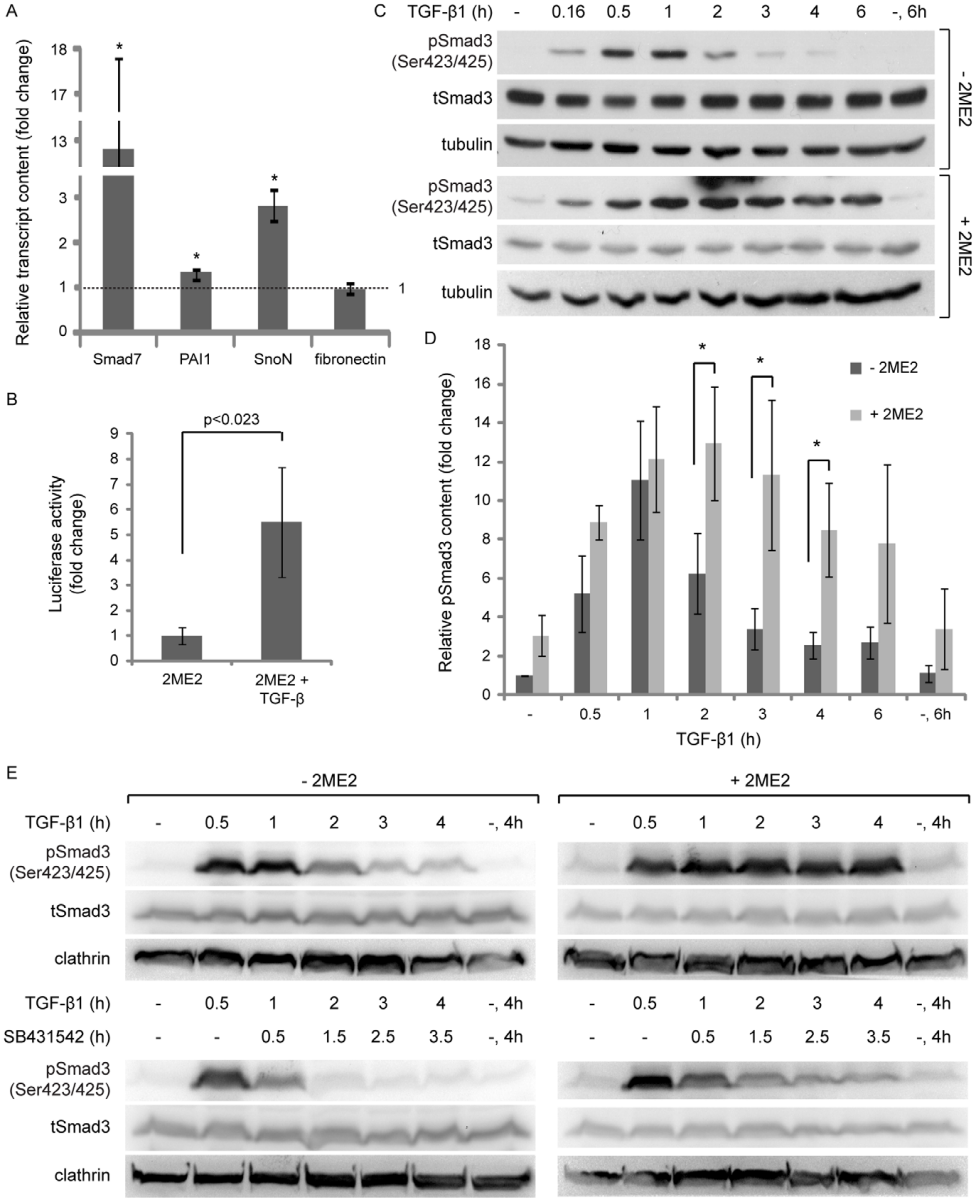
2ME2-arrest results in a prolongation of the TGF-β1-induced phosphorylation of Smad3. A, qRT-PCR analysis of Smad7, PAI-1, SnoN and fibronectin. Bar graph depicts the average ± SEM fold change in normalized transcript content in cultures treated with 2ME2 and stimulated with TGF-β1 (5 ng/ml, 2 h) as compared to cultures treated with 2ME2 alone. *, n = 6; p<0.05; all 2-tailed t-tests. B, Transcriptional activation assay. ES-2 cells were co-transfected with the (CAGA)_12_-Luc reporter construct and pRL-TK and arrested in mitosis with 2ME2. Arrested cells stimulated with TGF-β1 (5 ng/ml, 6 h) showed a 5.5±2.2 fold increase in normalized luciferase signal, n = 3 independent experiments (6 wells/treatment/experiment); p<0.023, 1-tailed t-test. C, α-pSmad3C, α-tSmad3 and α-tubulin immunoblots of ES-2 cells, treated with 2ME2 or vehicle, and stimulated with TGF-β1 for the indicated times. D, Bar graph depicts the average ± SEM fold increase in pSmad3C/tubulin ratio in cycling/untreated cells (dark grey bars) and in cells arrested with 2ME2 (light grey bars). 2ME2-mediated mitotic arrest significantly increased pSmad3C/tubulin ratio at 2, 3 and 4 hours of TGF-β1 stimulation, *, p<0.02; all with 2-tailed t-tests. E, α-pSmad3C, α-tSmad3 and α-clathrin immunoblots of ES-2 cells, treated with 2ME2 or vehicle and stimulated with TGF-β1. In indicated samples, SB431542 was added at 0.5 h post-TGF-β1 addition.

Having observed that the mitosis-induced receptor-independent phosphorylation of Smad3 does not generate a transcriptional response (Figure 2B–C), we next examined the signaling output of cells arrested in mitosis and stimulated with TGF-β1. In 2ME2-arrested cells, TGF-β1 induced a significant increase in the transcript levels of Smad7, SnoN, PAI-1, but not fibronectin (Figure 5A). Moreover, TGF-β1 also significantly activated the (CAGA)_12_-Luc reporter construct in 2ME2-arrested cells (Figure 5B). In contrast to the characteristic bell-shaped activation/de-activation profile of Smad3 observed with cycling ES-2 cultures upon continuous exposure to TGF-β1, cells arrested with 2ME2 presented sustained pSmad3C levels, even at 6 h after ligand addition (Figure 5C–D). Analogous results (a TGF-β-induced transcriptional response and sustained pSmad3C levels at late time points after ligand addition) were obtained with HEY cells arrested in mitosis (Figure S1A and C).

**Figure 6:**
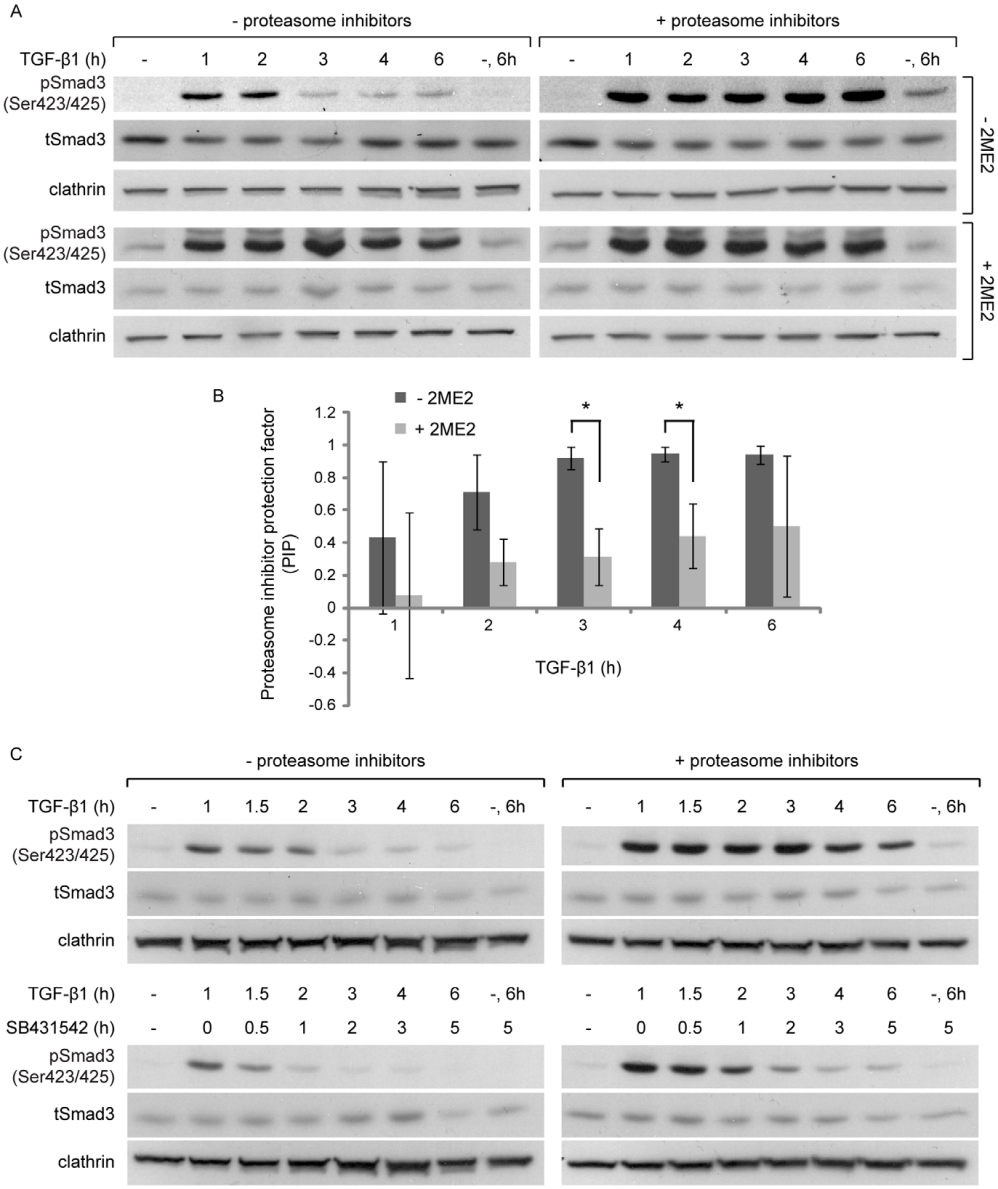
Proteasome inhibition prolongs the pSmad3 signal with a dependence on TβRI kinase activity. A, α-pSmad3C, α-tSmad3 and α-clathrin immunoblots of ES-2 cells, treated with 2ME2 or vehicle, proteasome inhibitors (MG132 and ALLN), or their combination, and stimulated with TGF-β1 for the indicated times. B, Bar graph depicts average ± SD of the proteasome inhibitor protection factor (PIP) at each time point after ligand addition. The factor is defined as PIP = 1−(pSmad3C/tSmad3/clathrin)_uninhibited_/(pSmad3C/tSmad3/clathrin)_inhibited_. n = 3; p<0.015, 1-tailed t-test. C, α-pSmad3C, α-tSmad3 and α-clathrin immunoblots of ES-2 cells, treated with proteasome inhibitors or vehicle and stimulated with TGF-β1. In indicated samples, SB431542 was added at 1 h after TGF-β1 stimulation.

**Figure 7:**
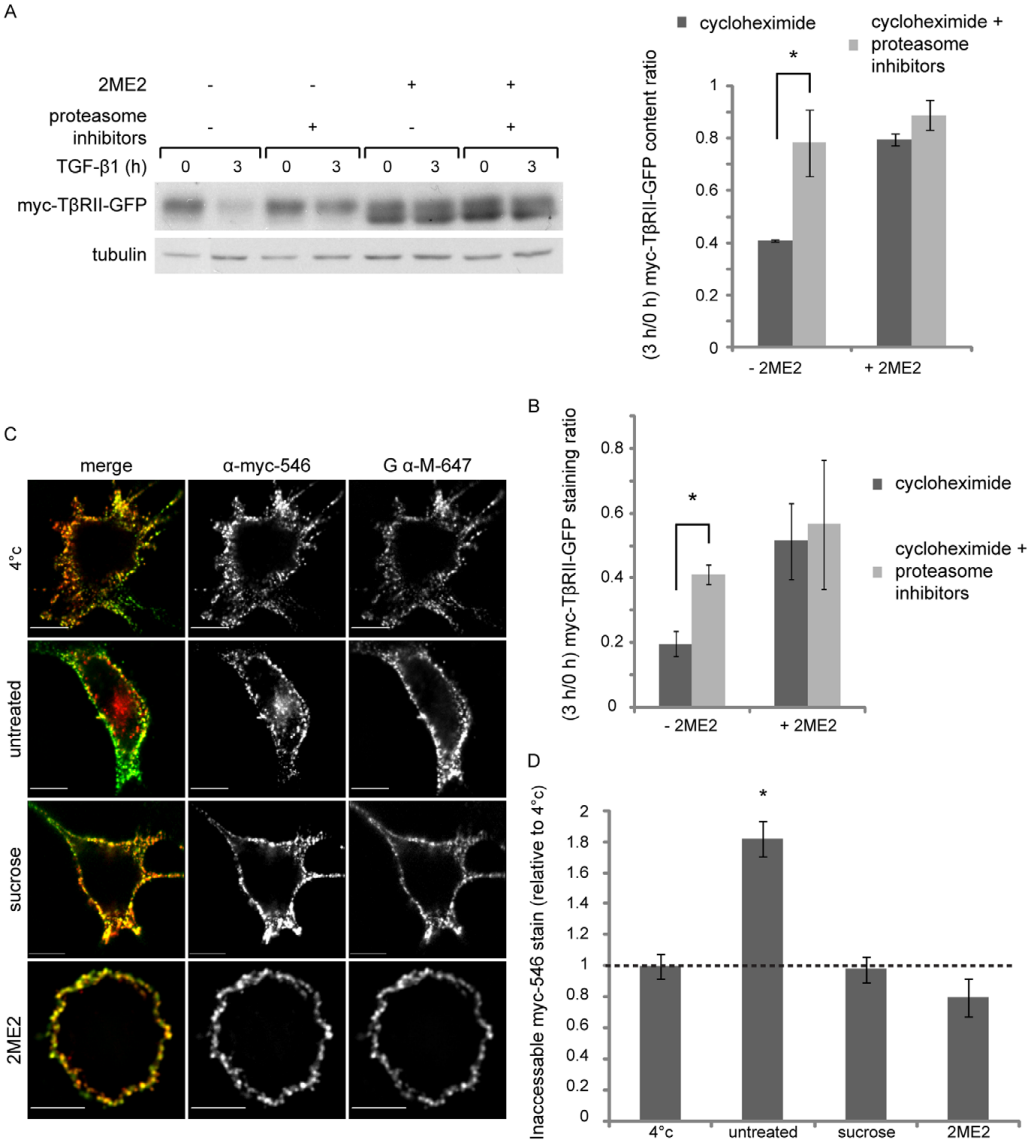
2ME2 impairs the turnover and endocytosis of myc-TβRII-GFP. A, ES-2 cells, stably expressing myc-TβRII-GFP, were treated with 2ME2 or vehicle. Arrested and cycling cells were pre-treated for 20 min with cycloheximide (300 µg/ml), in combination (or not) with proteasome inhibitors (MG132 and ALLN); followed by the addition of TGF-β1 (5 ng/ml, 3 h, in the same media). Cells were subsequently lysed and immunoblotted with α-myc antibodies. Bar graph depicts average ± SEM of the 3 h/0 h ratio of the relative myc-TβRII-GFP content, n = 3; p = 0.05, 2-tailed t-test. B, ES-2 cells grown on glass coverslips were treated as in (A), cooled to 4°C, stained with α-myc/Alexa-546-GαM/DAPI and imaged by confocal microscopy. Bar graph depicts average ± SEM of the 3 h/0 h ratio of the Alexa-546 staining normalized to the DAPI staining of 18 fields of cells (10 x magnification, ∼1400 cells) for each condition; p<0.01. C, Confocal micrographs of ES-2 cells, stably expressing myc-TβRII-GFP, treated with 2ME2, hypertonic medium (0.45 M sucrose, 30 min), or vehicle and submitted to endocytosis experiment (as described in the Materials and Methods). Bar, 10 µm. Specific signals were identified through intensity-based segmentation, and co-localized signal-positive pixels were determined with Slidebook™ and employed for quantification. D, Bar graph depicts the average fold change (± SEM) in the percent of 546-α-myc that did not co-localize with 647-GαM in all experimental conditions as compared to that of cells kept at 4°C. Cycling cells show a 1.82±0.11 fold increase in internalized TβRII (n = 35; p<0.01, 1-tailed t-test); no changes in the levels of internalized TβRII were observed upon hypertonic medium treatment (0.97±0.08 fold change, n = 8; p>0.8, 2-Tailed t-test) and a slight reduction of this parameter, which failed to reach statistical significance, was induced by arrest in mitosis with 2ME2 (0.8±0.12 fold change; n = 20 cells; p>0.16, 2-tailed t-test).

To better understand the cause of the sustained pSmad3C levels, at late time points after exposure to TGF-β1 in the 2ME2-arrested cells, we explored different lines of experimentation. (i) In the context of ES-2 cells arrested in mitosis with nocodazole, TGF-β1 induced sustained pSmad3C levels at late time points after ligand addition (Figure S5A). From this we conclude that the observed signal prolongation is mitosis-related and not restricted to 2ME2-treated cells. (ii) In contrast, in the context of cycling ES-2 cells subjected to a short nocodazole treatment, which depolymerizes microtubuli without inducing a cell cycle arrest, TGF-β1 induced a bell-shaped activation/de-activation profile of pSmad3C (Figure S5B). These data indicate that additional attributes of the mitotic cell, other than the absence of a polymerized microtubule network, are required for the sustained pSmad3C levels observed in the 2ME2-arrested cells. (iii) We probed for the putative contribution of continuous TGF-β receptor kinase activity in the generation of the sustained pSmad3C levels observed in cells arrested in mitosis. Addition of the kinase inhibitor SB431542 (30 min after stimulation with TGF-β1 of cells arrested in mitosis) resulted in a marked decrease in pSmad3C levels at later time points of TGF-β1 stimulation (Figure 5E). These data indicate that the reduction in pSmad3C levels, which can occur through de-phosphorylation or degradation, can still occur in the context of a mitotic cell, and suggest that the sustained pSmad3C levels observed in the cells arrested in mitosis stem, at least in part, from prolonged activity of the TGF-β receptor. However, the pSmad3C levels of cells arrested with 2ME2 and treated with SB431542 remained higher than those of their un-arrested counterparts. This suggests that in addition to the impaired down-regulation of TGF-β receptor activity in mitosis, additional mechanisms, such as the reduced activity of phosphatases, may also contribute to the sustained pSmad3C levels observed in this condition. (iv) We examined the role of the proteasome in mediating the termination of the TGF-β signal in cycling cells, and the putative perturbation of this mechanism in cells arrested in mitosis. Inhibition of proteasome activity markedly prolonged and enhanced the pSmad3C response in cycling ES-2 cells (Figure 6A, upper panels). In contrast, only a slight addition to the already prolonged pSmad3C signal could be observed upon proteasome inhibition in the 2ME2-arrested cells (Figure 6A, lower panels). To quantify the differential effects of proteasome inhibitors in cycling and arrested cells, we formulated and calculated a proteasome inhibitor protection factor (PIP; PIP = 1-(relative pSmad3C levels_uninhibited_/relative pSmad3C levels_inhibited_)). For all time points after ligand addition, the PIP value obtained with cycling cells was higher than the one observed in cells arrested with 2ME2 (Figure 6B). These results are in line with the impairment of a proteasome-mediated signal attenuation mechanism in mitosis. The sustained pSmad3C levels observed in cells treated with proteasome inhibitors may reflect either a continuous generation of new pSmad3C, or a lack of pSmad3C clearance through degradation or de-phosphorylation. To discern amongst these scenarios, we added SB431542 to cells treated with proteasome inhibitors (30 min after TGF-β1 addition). In these conditions, a substantial time-dependent decrease in pSmad3C levels was observed (Figure 6C), suggesting that proteasome activity regulates the generation of pSmad3C. However, the pSmad3C levels of cells treated with proteasome inhibitors and SB431542 remained higher than those treated with SB431542 alone. These data support the notion of an additional, albeit minor, proteasome-dependent mechanism of attenuation of pSmad3C levels that is not dependent on the kinase activity of the TGF-β receptor. (v) Next, we assayed the effects of arrest in mitosis and proteasome inhibition on the turnover of the type II TGF-β receptor (TβRII). To this end we generated an ES-2-based cell line stably expressing myc-TβRII-GFP (TβRII with a myc epitope at its N-terminus and fused to GFP at its C-terminus). Inhibition of protein synthesis with cycloheximide induced a significant reduction in myc-TβRII-GFP levels in cells stimulated with TGF-β1 (Figure 7A). Inhibition of the proteasome significantly countered this cycloheximide-induced reduction (Figure 7A). The decrease in myc-TβRII-GFP levels induced by cycloheximide was also markedly reduced in 2ME2-arrested cells, and a lesser effect was observed upon proteasome inhibition in these conditions (Figure 7A). Furthermore, imaging-based experiments aimed at following the cycloheximide-induced decrease in myc-TβRII-GFP levels at the cell surface revealed an identical picture. Namely, the reduction induced by cycloheximide was reversed by proteasome inhibition and by arrest in mitosis with 2ME2 (Figure 7B).

Our recent work points to a selective inhibition of clathrin-mediated internalization of receptors in mitosis [34]. To examine if the endocytosis of TβRII is altered in mitosis we employed an antibody feeding assay. Cycling or arrested cells were fed with Alexa-546-labeled monoclonal α-myc antibody (546-α-myc, 20–30 min, 37°C), cooled to 4°C and the cell-surface population of receptors was labeled with Alexa-647-goat-α-mouse antibody (647-GαM). Cells were imaged by confocal microscopy and the percentage of 546-α-myc signal that did not co-localize with the 647-GαM signal was calculated. To obtain a baseline value, we measured the lack of overlap of 546-α-myc and 647-GαM in conditions in which endocytosis does not occur (4°C), this value was employed in subsequent calculations of myc-TβRII-GFP endocytosis. After 30 min at 37°C, 46.2±2.9% of the 546-α-myc signal was inaccessible to 647-GαM, representing a 1.82±0.11 fold increase when compared to the value obtained at 4°C. Moreover, visual inspection of the confocal micrographs acquired in these conditions revealed the accumulation of 546-α-myc puncta at the cell interior (Figure 7C). To probe for the dependence of this internalization on a functional clathrin-mediated endocytic pathway, we performed two lines of experiments: (i) treatment with 0.45 M sucrose (hypertonic medium), a well established clathrin-endocytosis inhibitory treatment [50]; which blocked receptor internalization, as observed by the membrane-localized staining obtained for 546-α-myc and its high level of co-localization with 647-GαM staining (Figure 7D). (ii) siRNA-mediated knockdown of clathrin. In clathrin-depleted cells, the internalization of the receptor was blocked in the absence of TGF-β (Figure S6) or its presence (not shown). These data show that the clathrin-mediated endocytic pathway is the main internalization pathway employed by TβRII in ES-2 cells. Visualization of myc-TβRII-GFP internalization in 2ME2-arrested cells revealed a complete block in its endocytosis. Here, in addition to a lack of internal 546-α-myc puncta (Figure 7C–D), only 20±3% 546-α-myc was inaccessible to 647-GαM (0.44 fold of the value obtained with untreated cells at 37°C). Importantly, a similar lack of internalization was observed in randomly-selected cycling-mitotic cells (Figure S7). Taken together, these data support the notion that the clathrin-mediated internalization of TβRII is blocked in mitosis.

The role performed by endocytosis in TGF-β signaling is a contentious matter [22], [23], [24], [51]. The endocytosis of TβRII was proposed to be a primary determinant of Smad de-activation kinetics through the clearance of TGF-β from the medium [52]. To probe if the mitosis-induced inhibition of TβRII endocytosis results in a reduction in ligand clearance, we performed a 2 step medium transfer assay (see methods). Media from 2ME2-arrested cells retained a 2.6±0.6 fold higher activation potential then media from cycling cells (n = 7; p<0.025, 1-tailed t-test), suggesting that mitotic cell cultures are impaired in their ligand depletion capacity (Figure S8). This impairment of TGF-β clearance cannot be solely attributed to the reduction in cell number stemming from the 2ME2-arrest, as the expected increase in cell number of untreated cultures, at 16 h, would only be of 1.6 fold (Figure 1E). To examine the role of TβRII in this process, we measured the ligand depletion potential of ES-2 cells stably over-expressing myc-TβRII-GFP. These cells cleared a greater amount of ligand from the medium, as the signal-activating potential of their medium was 0.6±0.06 fold of untransfected ES-2 (n = 3; p<0.035, 1-tailed t-test). These data support the notion that TβRII endocytosis depletes ligand from the medium, and that this mechanism is reduced in mitosis. To assess if a block in clathrin mediated endocytosis alters the activation or attenuation parameters of TGF-β signaling in cycling ES-2 cells, we examined the intracellular distribution of Smad3 in cells knocked-down, or not, for α-adaptin or clathrin heavy chain, stimulated or not with TGF-β1 (for 1 h), and incubated with fluorescent transferrin in the final 10 min of the TGF-β stimulation. Inhibition of clathrin mediated endocytosis did not affect the ability of TGF-β1 to induce the nuclear translocation of Smad3 (Figures S9,S10). However, depletion of α-adaptin or clathrin did not affect the pSmad3C attenuation kinetics (Figures S9,S10). Also, treatment of ES-2 cells with β-cyclodextrin, which reduces the cholesterol content of cells and blocks clathrin-independent internalization pathways, was also devoid of effects on the profile of attenuation of Smad3 phosphorylation (Figure S11). In summary, our data point to an impairment of a proteasome-dependent mechanism of attenuation of the TGF-β receptor signaling in mitotic cells, and to the localization of this receptor attenuation step to the plasma membrane, at least in cells in which endocytosis has been blocked.

## Discussion

The mitotic cell is characterized by dramatic changes to cell state, which include a temporary reduction in cell volume and a concomitant condensation of the cytosol [48], [49], a selective inhibition of receptor-mediated endocytosis [30], [34], a mitotic stage-specific abrogation of endosomal recycling [30], a reorganization of tubulin to the mitotic spindle, the activation of mitotic kinases such as Mps1 [44], [53], and of kinases such as ERK [54], [55]. Notably, endocytosis [22], [23], [24], [51], [56], [57], [58], recycling [59], Mps1 [37], ERK [38], [60], microtubules and microtubule-associated proteins [4], [46], have all been implicated in the regulation of TGF-β/Smad signaling; suggesting that multiple aspects of the regulation of the TGF-β signal may be altered in mitosis. Indeed, the regulation of TGF-β and Smad signaling in mitosis has been recently studied in different cellular models [20], [37], [61]. These studies showed that the cellular interpretation to TGF-β stimuli (induction of epithelial to mesenchymal transition or apoptosis) is cell cycle dependent in AML-12 cells [61], Smad3 levels are higher in quiescent mouse mammary gland epithelial cells and drop in proliferating cells [20], Smads 2 and 3 are activated by the mitotic kinase Mps1 in the absence of ligand stimulation in a number of cell models [37], and Smad3 associates with its negative regulators Ski and SnoN in mitosis [15].

Here, we employed mesenchymal-like ovarian cancer cells as a cellular model and 2-methoxyestradiol (2ME2) as a mitosis-arresting agent and showed that in cells arrested at the spindle assembly checkpoint with 2ME2 (i) Smad3 is phosphorylated at its C-terminus and threonine 179 in a manner that is independent of the kinase activity of TβRI, (ii) the Smad3 cellular content is reduced, (iii) the receptor-independent phosphorylation of Smad3 does not induce a transcriptional response, (iv) pSmad3C preferentially associates with Ski and Smurf2; and (v) pSmad3C accumulates upon proteasome inhibition. We also show that following TGF-β stimulation of cells arrested in mitosis (vi) signal attenuation is compromised and sustained levels of pSmad3C are observed even at 4–6 hours after TGF-β addition. Moreover, we observed that (vii) the clathrin-mediated endocytosis of the type II TGF-β receptor (TβRII) is blocked in mitosis and (viii) its proteasome-mediated clearance is decreased. These findings are summarized schematically in Figure S12.

The notion of the coupling of Smad3 phosphorylation and the reduction of its levels in cells arrested in mitosis (in the absence of TGF-β stimulation) is supported by the following lines of evidence: (i) both the reduction in levels and the phosphorylation are inhibited by a specific inhibitor of Mps1 [44], by the incubation of the arrested cells in hypotonic medium, and by inhibition of ERK activation with U0126; (ii) proteasome inhibition in cells arrested in mitosis leads to a marked accumulation of pSmad3C. Notably, we also observed a reversine-sensitive C-terminus phosphorylation of over-expressed GFP-Smad3, suggesting that Mps1 can phosphorylate Smad3 in the context of interphase cells. ES-2 and HEY ovarian cancer cells are characterized by hyper-activating mutations in the B-Raf oncogene [39]; constitutively active B-Raf (V600E) interacts with, stabilizes and hyper-activates Mps1 in melanoma cells [62]; thus, ES-2 and HEY cells may be particularly sensitive to Mps1-mediated regulation of Smad3. The phosphorylations of the C-terminus and linker regions of receptor activated Smads dictate their repertoire of protein-protein interactions, influencing in this manner their activity and turnover. In this context, linker domain phosphorylation was proposed to mediate interactions with ubiquitin ligases [6], [7], [9]. Pin1, a peptidyl-prolyl cis/trans isomerase, was also proposed as a regulator of Smad2/3 turnover [7]. The binding site of Pin1 to Smad3 is phospho-threonine 179; however, phosphorylation of Smad3 at its C-terminus is also required for Smad3-Pin1 interactions [10]. In the present study, we identify the phosphorylation of Smad3 on both sites (threonine 179 and the C-terminus) in cells arrested in mitosis. We propose that these phosphorylations of Smad3 are connected to the reduction in its levels in mitotic cells.

TGF-β receptors are also a target for proteasome-mediated degradation [26], [27], [28], [63]. The HECT family of E3 ubiquitin ligases (Smurfs 1 and 2, Nedd4-2 and WWP1) were proposed to play a central role in the attenuation of the TGF-β signal [64]. Similarly to the controversy on the role of endocytosis on the transduction of the TGF-β signal [22], [23], [24], [51], the intracellular localization and mechanism of receptor attenuation, including the requirement for internalization and the putative route of entry employed for this process, are all contentious matters [22], [23], [24]. Here we show that in mesenchymal-like ovarian cancer cells, the activation and nuclear translocation of Smad3 do not depend on clathrin mediated endocytosis. Moreover, the TGF-β-induced transcriptional activation of target genes and of the (CAGA)_12_-Luc reporter gene construct which are observed in mitotic cells (a condition in which the endocytosis of TβRII is blocked), confirm the lack of a necessity of TβRII internalization for its signal transduction. Of note, the attenuation of the TGF-β signal, which yielded a bell-shaped profile of Smad3 phosphorylation in cycling ES-2 and HEY cells, was still observed when clathrin mediated endocytosis was blocked via the siRNA-mediated knockdown of clathrin or α-adaptin. Since the internalization of TβRII is exclusively via clathrin in ES-2 cells, and TβRII and TβRI form stable complexes in the presence of TGF-β [1], these data suggest a plasma-membrane localized mechanism of attenuation of TGF-β receptor activity in cells in which clathrin mediated endocytosis has been blocked. The current study falls short of determining if such a membrane-localized mechanism is present in unperturbed cells or if it is a result of the endocytic block, which may mislocalize regulatory factors involved in the attenuation of the TGF-β signal to the plasma membrane. The attenuating effect of SB431542 on the increase in pSmad3C, observed upon the inhibition of the proteasome in cells activated with TGF-β, supports the view that the activated receptors constitute an important target of the proteasome in the reduction of the TGF-β signal in ES-2 cells. In accord with this notion, proteasome inhibition reduced the clearance of TβRII from the plasma membrane. In cells arrested in mitosis, the maintenance of TβRII at the plasma membrane is correlated with a prolongation in the ligand-induced phosphorylation of Smad3 and with a lack of degradation of TβRII. Moreover, when compared to cycling cells, proteasome inhibition in mitotic cells induces lesser effects on both the accumulation of ligand-induced pSmad3C and on the accumulation of TβRII. These lesser effects suggest that the proteasome-mediated mechanism of attenuation of TGF-β receptor activity is hampered in mitotic cells. In addition to the possible segregation to different cellular compartments of receptors and degradation mediators, this hampering may also stem from the altered regulation of specific ubiquitin ligases in mitosis. For example, Nedd4-2 mediates the degradation TβRI [26]; Nedd4-2 activity is negatively regulated by phosphorylation on residues flanking its WW2 domain (Ser342 and Ser448) [65]; and the serine residues (Ser325, Ser327, Ser328, Ser330 and Ser343) were shown to be phosphorylated in cells arrested in mitosis [35]. Thus, mechanisms such as the putative negative regulation of Nedd4-2 in mitosis may contribute to the differential regulation of the TGF-β receptors.

### Speculative Model

We speculate that the retention of TβRII at the plasma membrane is necessary for the maintenance of the sensitivity of the mitotic cell to TGF-β stimulation. Our speculation is based on the combined perturbations to proteins synthesis [66] and endosomal recycling [30], which were proposed to occur in mitosis. In these conditions, in the absence of retention at the plasma membrane, a marked decrease in the membrane content of TβRII would be expected to occur in mitosis. Indeed, such depletion has been described for the transferrin receptor which is endocytosed, but not recycled, in mitosis [30]. In addition, we speculate that the proteasome-mediated negative regulation of Smad3 prior to exposure to TGF-β, and the attenuation of the proteasome-mediated negative regulation of the TGF-β receptor activity upon ligand activation, compensate for one another and allow for the maintenance of similar levels of cellular sensitivity to TGF-β stimulation in mitosis. What may be the importance of such a mechanism? Ligands of the TGF-β superfamily generate gradients of functional importance in development [67]. In these contexts, a regulated response to differing concentrations of ligand is expected to be important for the maintenance of positional identity while undergoing cell division.

## Supporting Information

Figure S1 **2ME2 augments pSmad3C levels following TGF-β stimulation and decreases tSmad3 levels in HEY cells.** A, α-pSmad3C, α-tSmad3 and α-tubulin immunoblot of HEY cells, arrested in mitosis with 2ME2 or treated with vehicle and stimulated with TGF-β1 for the indicated periods of time. B, α-tSmad3 and α-tubulin immunoblot of HEY cells treated with 2ME2 (16 h, 4.4 µM) or vehicle. Upon 2ME2-arrest the tSmad3/tubulin ratio was significantly reduced. C, qRT-PCR of Smad7. Bar graph depicts the average ± SEM fold change in normalized Smad7 transcript content upon TGF-β1 stimulation (5 ng/ml, 2 h) in cells treated with 2ME2 (16 h, 4.4 µM) or vehicle. TGF-β1 induced a 7.95±1.95 fold increase in Smad7 transcript content in cycling HEY cells, and a 5.12±3.2 fold increase in 2ME2-arrested cells, while no significant increase is observed upon the mitotic arrest alone.
(TIF)

Figure S2 **Dorsomorphin and A83-01 do not inhibit the phosphorylations of Smad3 in mitosis.** A, α-pSmad1/5/8, α-tSmad1/5/8, α-pSmad3C, α-tSmad3 and α-clathrin immunoblot of ES-2 cells, treated with 2ME2, dorsomorphin, their combination or vehicle. B, α-pSmad3C, α-pSmad3(179), α-tSmad3 and α-clathrin immunoblot of ES-2 cells, treated with 2ME2, A83-01, their combination or vehicle.
(TIF)

Figure S3 **U0126 counters the 2ME2-induced phosphorylation and decrease in levels of Smad3.** A, α-pSmad3C, α-pSmad3(179), α-tSmad3, α-pERK, α-tERK and α-clathrin immunoblot of ES-2 cells, treated with 2ME2, U0126, their combination or vehicle. B, ES-2 cells were treated with 2ME2, U0126, their combination or vehicle. DNA content was measured by propidium iodide staining and fluorescence-activated cell sorting (FACS).
(TIF)

Figure S4 **Reduction in Smurf2 is devoid of marked effects on the mitotic degradation of Smad3.** α-pSmad3C, α-tSmad3, α-Smurf2 and α-clathrin immunoblot of ES-2 cells, transfected with siRNA against Smurf2, with non-targeting siRNA or left untransfected and treated with 2ME2 or vehicle.
(TIF)

Figure S5 **Prolongation of the TGF-β1-induced phosphorylation of Smad3 upon G2/M arrest with nocodazole.** A, α-pSmad3C, α-tSmad3 and α-clathrin immunoblot of cells, treated with nocodazole (16 h) or vehicle and stimulated with TGF-β1. B, α-pSmad3C, α-tSmad3 and α-clathrin immunoblot of cells treated with nocodazole (1 h) or vehicle and stimulated with TGF-β1.
(TIF)

Figure S6 **The endocytosis of myc-TβRII-GFP is arrested in cells depleted of clathrin heavy chain.** Panels depict typical confocal micrographs of ES-2 cells, stably expressing myc-TβRII-GFP, transfected with siRNA against clathrin heavy chain or with non-targeting siRNA, and submitted to the antibody-feeding endocytosis assay (as described in Materials and Methods). Note the internalized 546-α-myc (apparent as red-labeled structures in the merged image of the cell transfected with control siRNA) as opposed to its absence in the cell depleted for clathrin heavy chain. Numbers depicts the percentage of cells positive for TβRII endocytosis (cells presenting more than 20% of 546-α-myc signal that did not co-localize with the 647-GαM signal), n = ∼25 cells; p<0.03 (2-tailed t-test).
(TIF)

Figure S7 **The endocytosis of myc-TβRII-GFP is arrested in cells undergoing unperturbed mitosis.** Panels depict confocal micrographs (single confocal plane, upper row; 3D rendition lower row) of ES-2 cells, stably expressing myc-TβRII-GFP, fed with alexa-546-anti-Myc antibodies (30 min, 37°C), cooled and labeled with alexa-647-labeled goat-anti-mouse antibodies (pseudo-colored in green, at 4°C). Note the internalized 546-α-myc (apparent as red labeled structures in the left cell in the merged image) as opposed to its absence in the interior of the mitotic cell.
(TIF)

Figure S8 **Arrest in mitosis with 2ME2 reduces clearance of TGF-β1 from medium.** Cells (referred as “donor cultures”) were arrested in mitosis with 2ME2 or treated with vehicle, and stimulated with TGF-β1 (3 h) or vehicle. Media, collected from these cultures, were transferred to naïve cells (referred as “reporter cultures”), for 1 h of stimulation (as described in Materials and Methods). Panels depict α-pSmad3C, α-tSmad3 and α-clathrin immunoblots of the reporter cultures. Bar graph depicts average ± SEM of the fold increase in pSmad3C/tSmad3 ratio in the lysates of the reporter cultures (media collected from 2ME2-arrested-TGF-β1-stimulated cells, as compared to media collected from non-arrested-TGF-β1-stimulated cells; 2.63±0.65 fold increase, n = 7; *, p<0.023, 1-tailed t-test).
(TIF)

Figure S9 **siRNA-mediated depletion of clathrin heavy chain does not block the phosphorylation and nuclear translocation of Smad3 or the attenuation of the TGF-β signal.** A, Confocal micrographs of ES-2 cells, transfected with siRNA against clathrin heavy chain or with non-targeting siRNA, activated or not with TGF-β1 (1 h), incubated with fluorescent transferrin (10 min, 100 µg/ml), and stained for clathrin heavy chain and Smad3. B, α-pSmad3C, α-tSmad3, α-clathrin and α-tubulin immunoblot of ES-2 cells, transfected with siRNA against clathrin heavy chain or with non-targeting siRNA and stimulated with TGF-β1 for the indicated times. C, Bar graph depicts the average ± SEM of the pSmad3C/tSmad3/tubulin signal (n = 3, no significant differences were observed between the clathrin depleted and the control cells).
(TIF)

Figure S10 **siRNA-mediated depletion of α-adaptin does not block the phosphorylation and nuclear translocation of Smad3 or the attenuation of the TGF-β signal.** A, Confocal micrographs of ES-2 cells, transfected with siRNA against α-adaptin or with non-targeting siRNA, activated or not with TGF-β1 (1 h), incubated with fluorescent transferrin (10 min, 100 µg/ml), and stained for α-adaptin and Smad3. B, α-pSmad3C, α-tSmad3 α- α-adaptin, and α-clathrin immunoblot of ES2 cells, transfected with siRNA against α-adaptin or with non-targeting siRNA and stimulated with TGF-β1 for the indicated times. C, Bar graph depicts the average ± SEM of the pSmad3C/tSmad3/tubulin signal (n = 3, no significant differences were observed between the α-adaptin depleted and the control cells).
(TIF)

Figure S11 **Reduction of cholesterol content does not alter the profile of attenuation of Smad3 phosphorylation.** α-pSmad3C, α-tSmad3 and α-clathrin immunoblots of ES-2 cells, treated with β-cyclodextrin (5 mM) or vehicle, and stimulated with TGF-β1 for the indicated times.
(TIF)

Figure S12 **Schematic depiction of the mitosis-induced alterations to Smad3 and TGF-β receptor signaling.** A, In interphase cells, TGF-β receptors endocytose and recycle constitutively. Upon TGF-β stimulation, Smad3 is phosphorylated at its C-terminus SSXS motif. Signal attenuation occurs through the proteasome-mediated down-regulation of the signaling receptor complex. B, In un-stimulated mitotic cells, Smad3 is phosphorylated at its C-terminus and threonine 179 in a ligand/receptor -independent fashion and localizes to vicinity of the mitotic spindle. In these conditions, Smad3 binds Smurf2 and Ski and fails to induce a transcriptional response. Moreover, in mitosis a reduction of Smad3 levels is observed. Furthermore, in mitosis, recycling in general and the endocytosis of the TGF-β receptors in particular, are arrested. The endocytosis arrest allows for the maintenance of TGF-β receptors at the plasma membrane. TGF-β-stimulation of mitotic cells induces a transcriptional response through a prolonged phosphorylation of pSmad3C, due to the reduced proteasome-mediated de-activation/degradation of the receptors.
(TIF)
