# Supplementary figures and images for: Differential Regulation of Smad3 and of the Type II Transforming Growth Factor-β Receptor in Mitosis: Implications for Signaling
Source: PLoS One. 2012 Aug 22;7(8):e43459. doi: 10.1371/journal.pone.0043459 (PMC3425481; doi:10.1371/journal.pone.0043459)

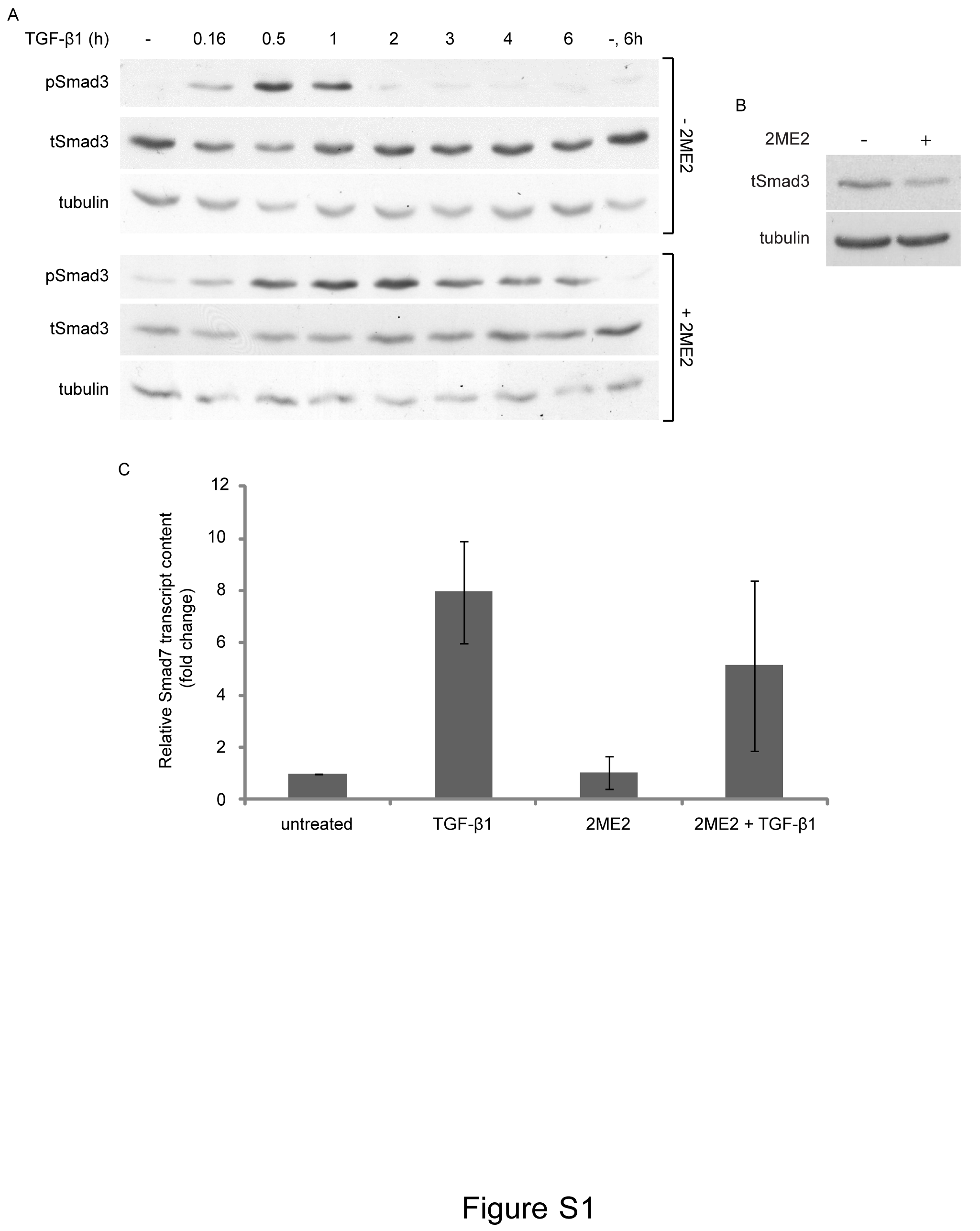

Supplement: Figure S1 — 2ME2 augments pSmad3C levels following TGF-β stimulation and decreases tSmad3 levels in HEY cells. A, α-pSmad3C, α-tSmad3 and α-tubulin immunoblot of HEY cells, arrested in mitosis with 2ME2 or treated with vehicle and stimulated with TGF-β1 for the indicated periods of time. B, α-tSmad3 and α-tubulin immunoblot of HEY cells treated with 2ME2 (16 h, 4.4 µM) or vehicle. Upon 2ME2-arrest the tSmad3/tubulin ratio was significantly reduced. C, qRT-PCR of Smad7. Bar graph depicts the average ± SEM fold change in normalized Smad7 transcript content upon TGF-β1 stimulation (5 ng/ml, 2 h) in cells treated with 2ME2 (16 h, 4.4 µM) or vehicle. TGF-β1 induced a 7.95±1.95 fold increase in Smad7 transcript content in cycling HEY cells, and a 5.12±3.2 fold increase in 2ME2-arrested cells, while no significant increase is observed upon the mitotic arrest alone. (TIF) [file pone.0043459.s001.tif]

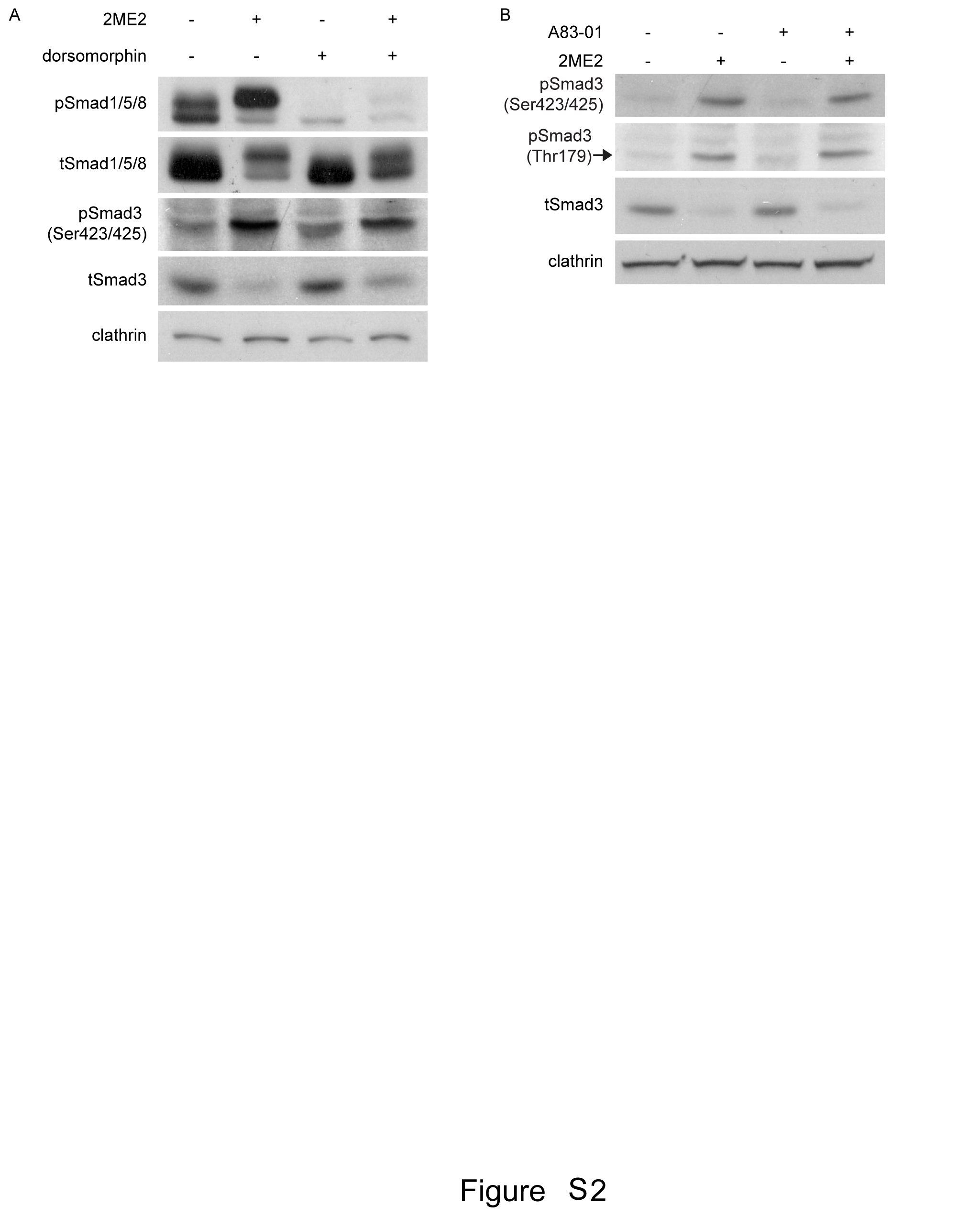

Supplement: Figure S2 — Dorsomorphin and A83-01 do not inhibit the phosphorylations of Smad3 in mitosis. A, α-pSmad1/5/8, α-tSmad1/5/8, α-pSmad3C, α-tSmad3 and α-clathrin immunoblot of ES-2 cells, treated with 2ME2, dorsomorphin, their combination or vehicle. B, α-pSmad3C, α-pSmad3(179), α-tSmad3 and α-clathrin immunoblot of ES-2 cells, treated with 2ME2, A83-01, their combination or vehicle. (TIF) [file pone.0043459.s002.tif]

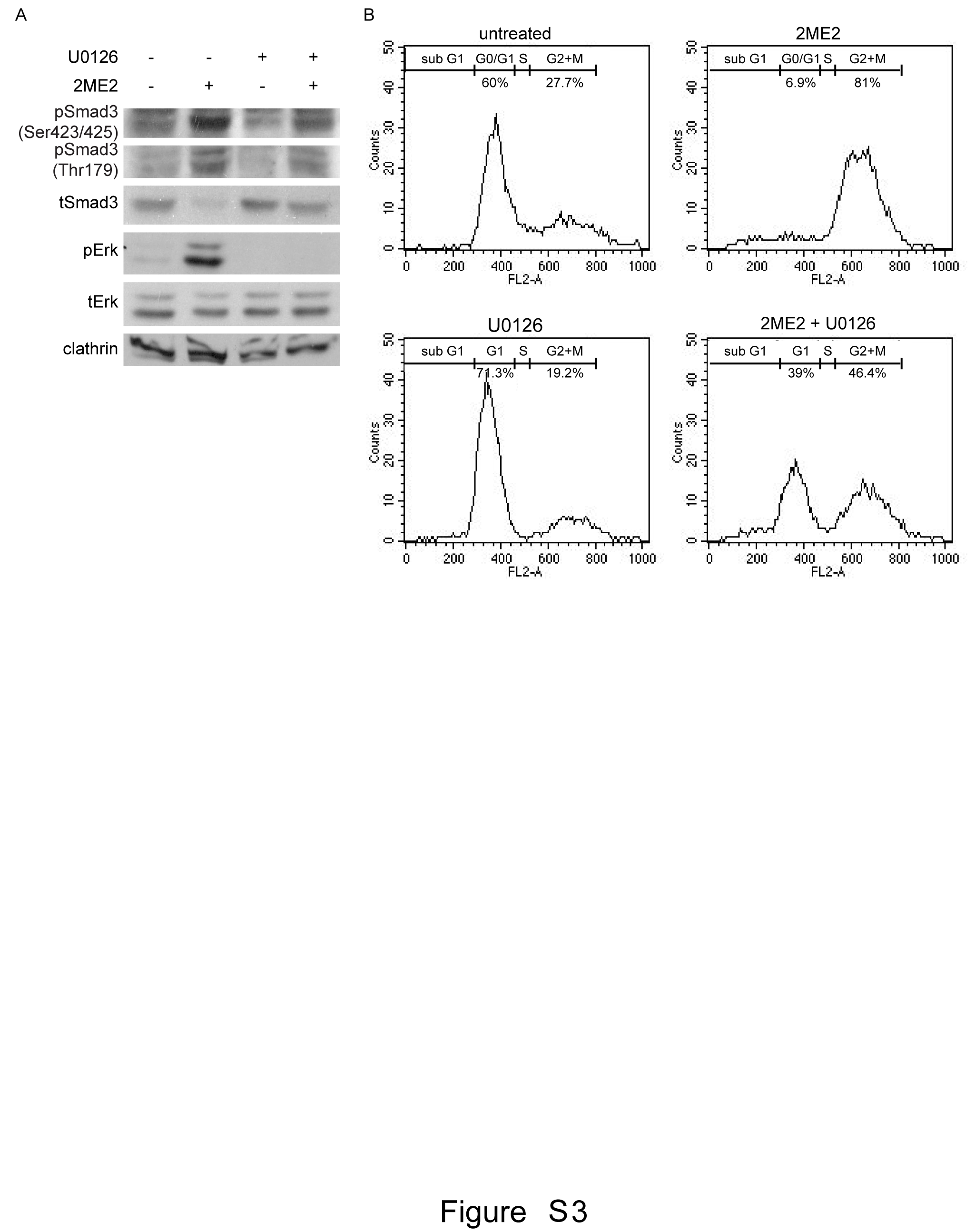

Supplement: Figure S3 — U0126 counters the 2ME2-induced phosphorylation and decrease in levels of Smad3. A, α-pSmad3C, α-pSmad3(179), α-tSmad3, α-pERK, α-tERK and α-clathrin immunoblot of ES-2 cells, treated with 2ME2, U0126, their combination or vehicle. B, ES-2 cells were treated with 2ME2, U0126, their combination or vehicle. DNA content was measured by propidium iodide staining and fluorescence-activated cell sorting (FACS). (TIF) [file pone.0043459.s003.tif]

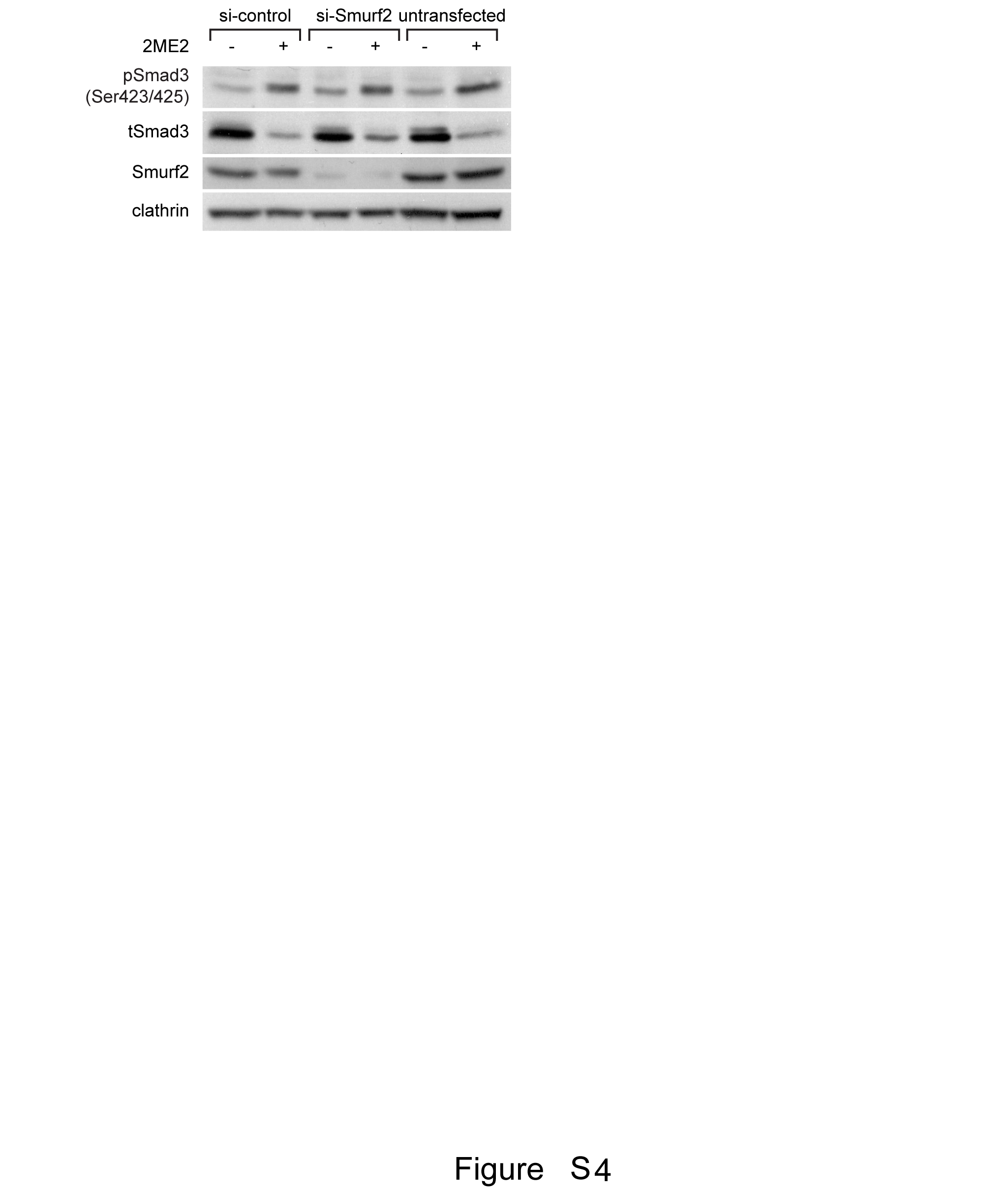

Supplement: Figure S4 — Reduction in Smurf2 is devoid of marked effects on the mitotic degradation of Smad3. α-pSmad3C, α-tSmad3, α-Smurf2 and α-clathrin immunoblot of ES-2 cells, transfected with siRNA against Smurf2, with non-targeting siRNA or left untransfected and treated with 2ME2 or vehicle. (TIF) [file pone.0043459.s004.tif]

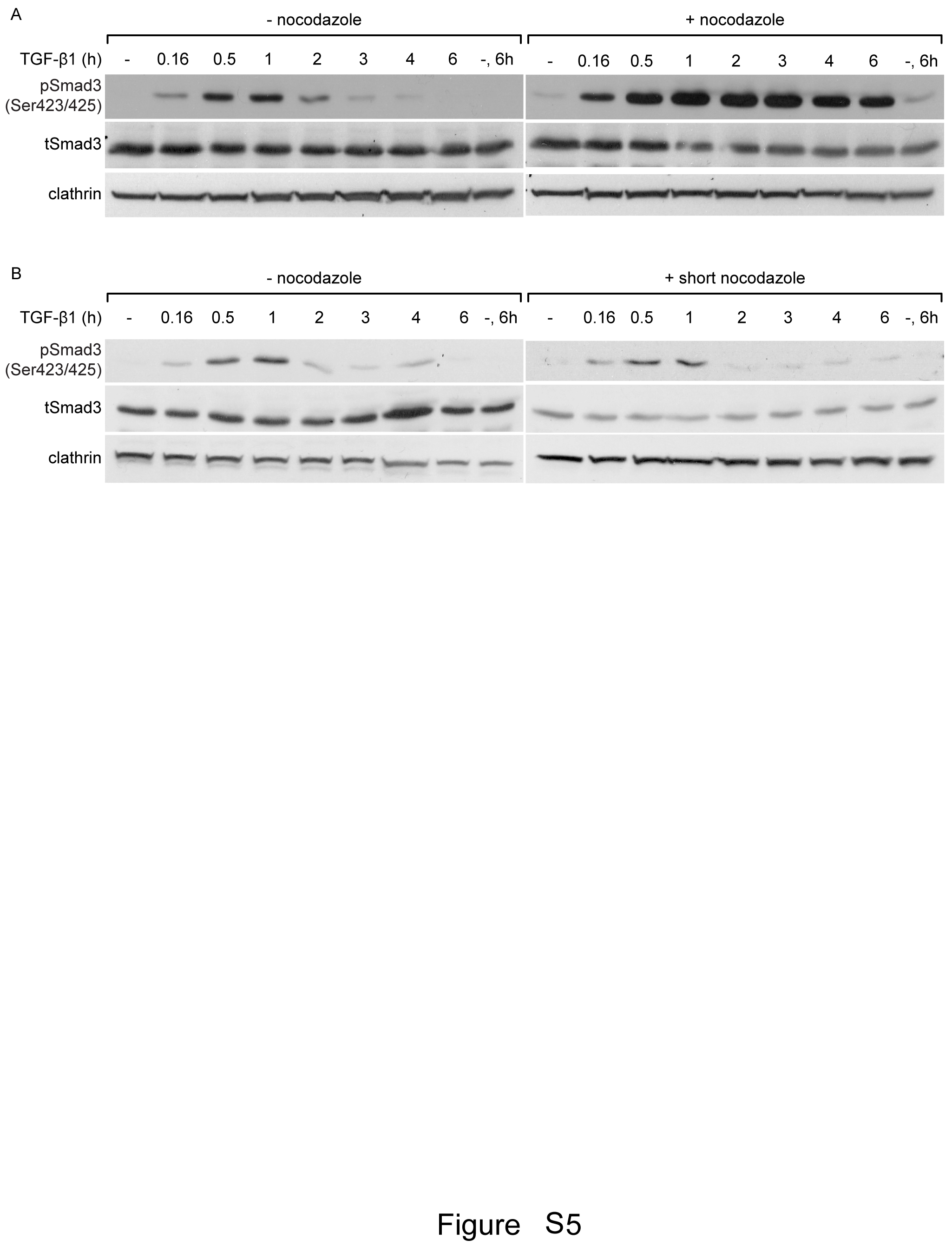

Supplement: Figure S5 — Prolongation of the TGF-β1-induced phosphorylation of Smad3 upon G2/M arrest with nocodazole. A, α-pSmad3C, α-tSmad3 and α-clathrin immunoblot of cells, treated with nocodazole (16 h) or vehicle and stimulated with TGF-β1. B, α-pSmad3C, α-tSmad3 and α-clathrin immunoblot of cells treated with nocodazole (1 h) or vehicle and stimulated with TGF-β1. (TIF) [file pone.0043459.s005.tif]

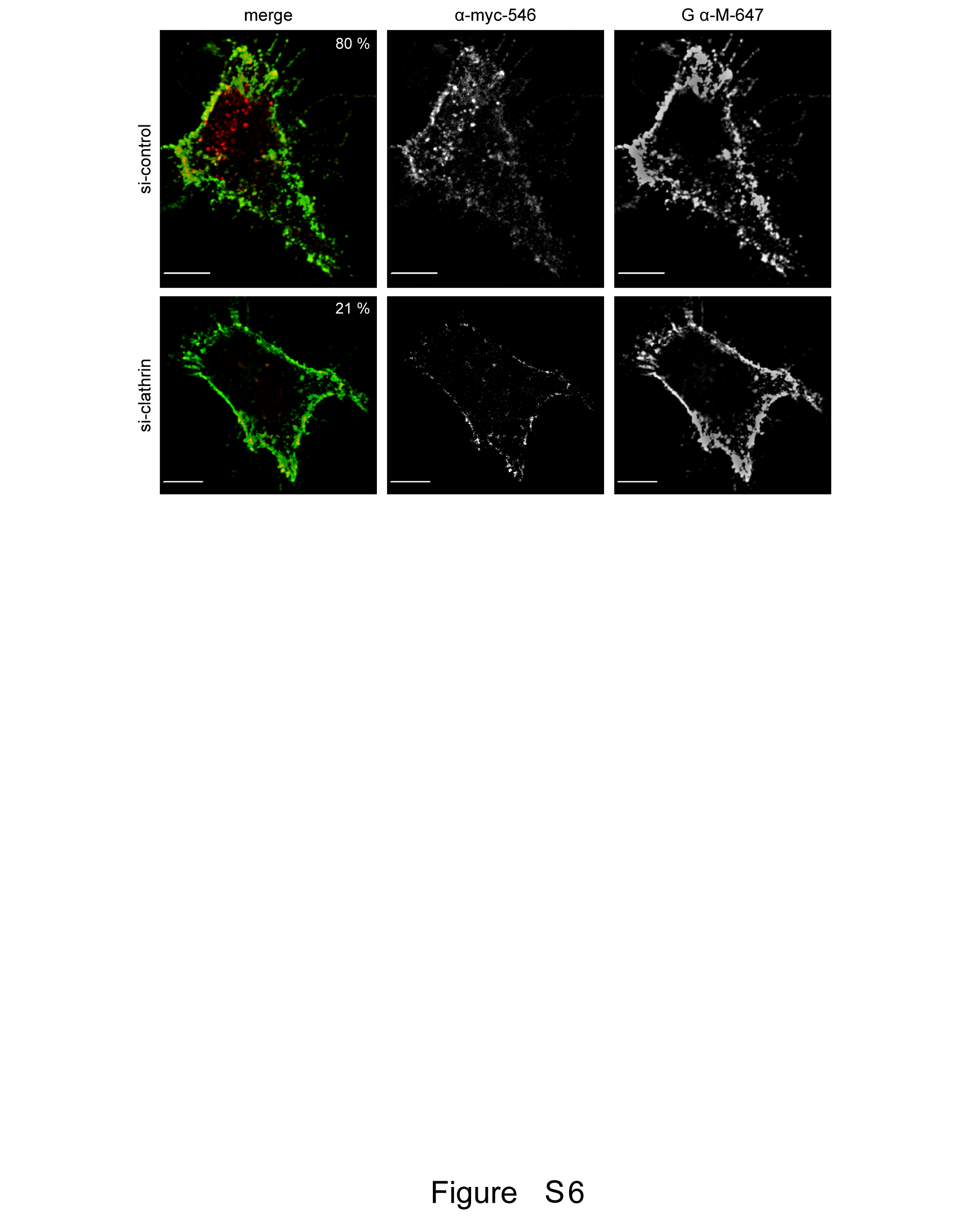

Supplement: Figure S6 — The endocytosis of myc-TβRII-GFP is arrested in cells depleted of clathrin heavy chain. Panels depict typical confocal micrographs of ES-2 cells, stably expressing myc-TβRII-GFP, transfected with siRNA against clathrin heavy chain or with non-targeting siRNA, and submitted to the antibody-feeding endocytosis assay (as described in Materials and Methods). Note the internalized 546-α-myc (apparent as red-labeled structures in the merged image of the cell transfected with control siRNA) as opposed to its absence in the cell depleted for clathrin heavy chain. Numbers depicts the percentage of cells positive for TβRII endocytosis (cells presenting more than 20% of 546-α-myc signal that did not co-localize with the 647-GαM signal), n = ∼25 cells; p<0.03 (2-tailed t-test). (TIF) [file pone.0043459.s006.tif]

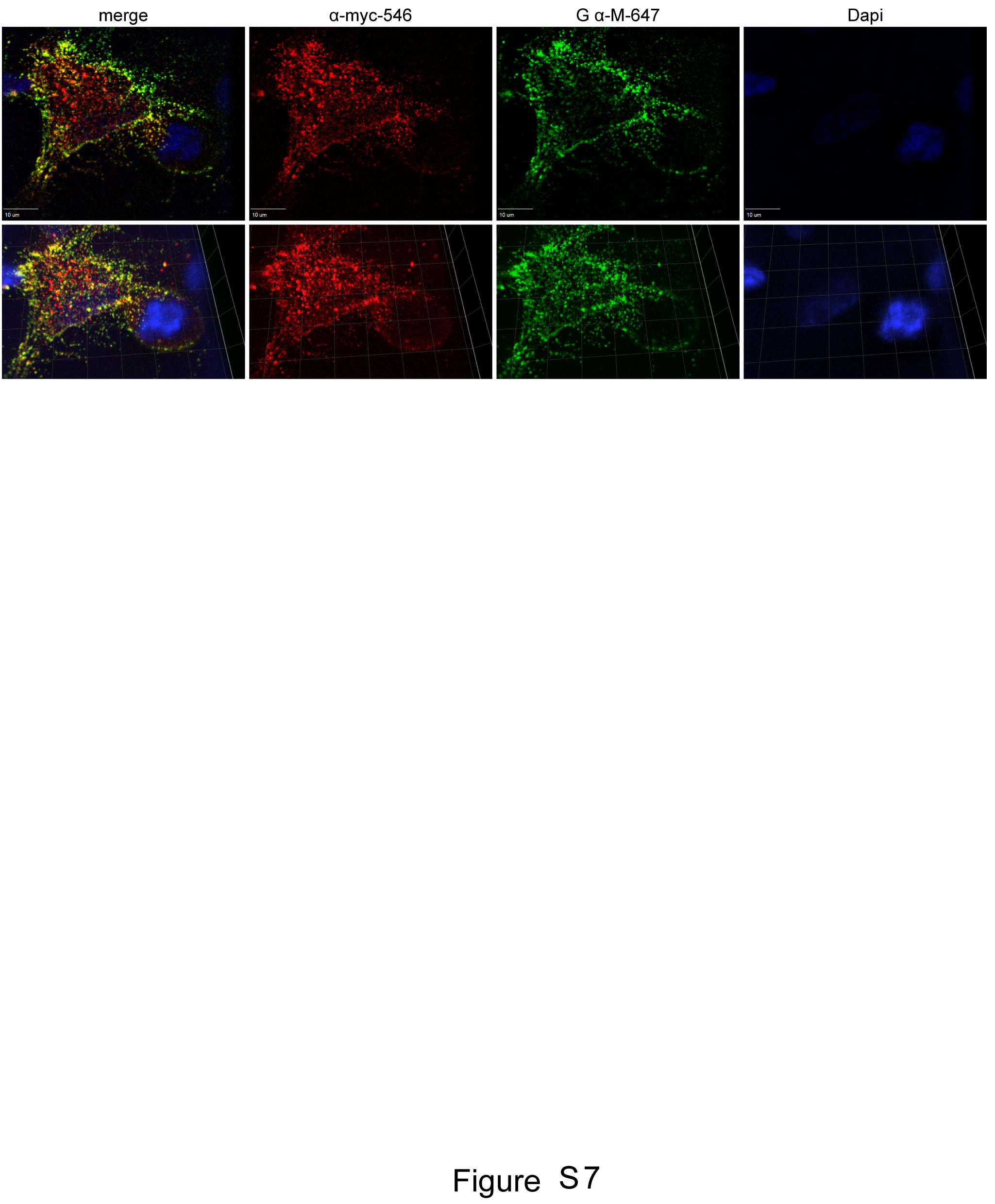

Supplement: Figure S7 — The endocytosis of myc-TβRII-GFP is arrested in cells undergoing unperturbed mitosis. Panels depict confocal micrographs (single confocal plane, upper row; 3D rendition lower row) of ES-2 cells, stably expressing myc-TβRII-GFP, fed with alexa-546-anti-Myc antibodies (30 min, 37°C), cooled and labeled with alexa-647-labeled goat-anti-mouse antibodies (pseudo-colored in green, at 4°C). Note the internalized 546-α-myc (apparent as red labeled structures in the left cell in the merged image) as opposed to its absence in the interior of the mitotic cell. (TIF) [file pone.0043459.s007.tif]

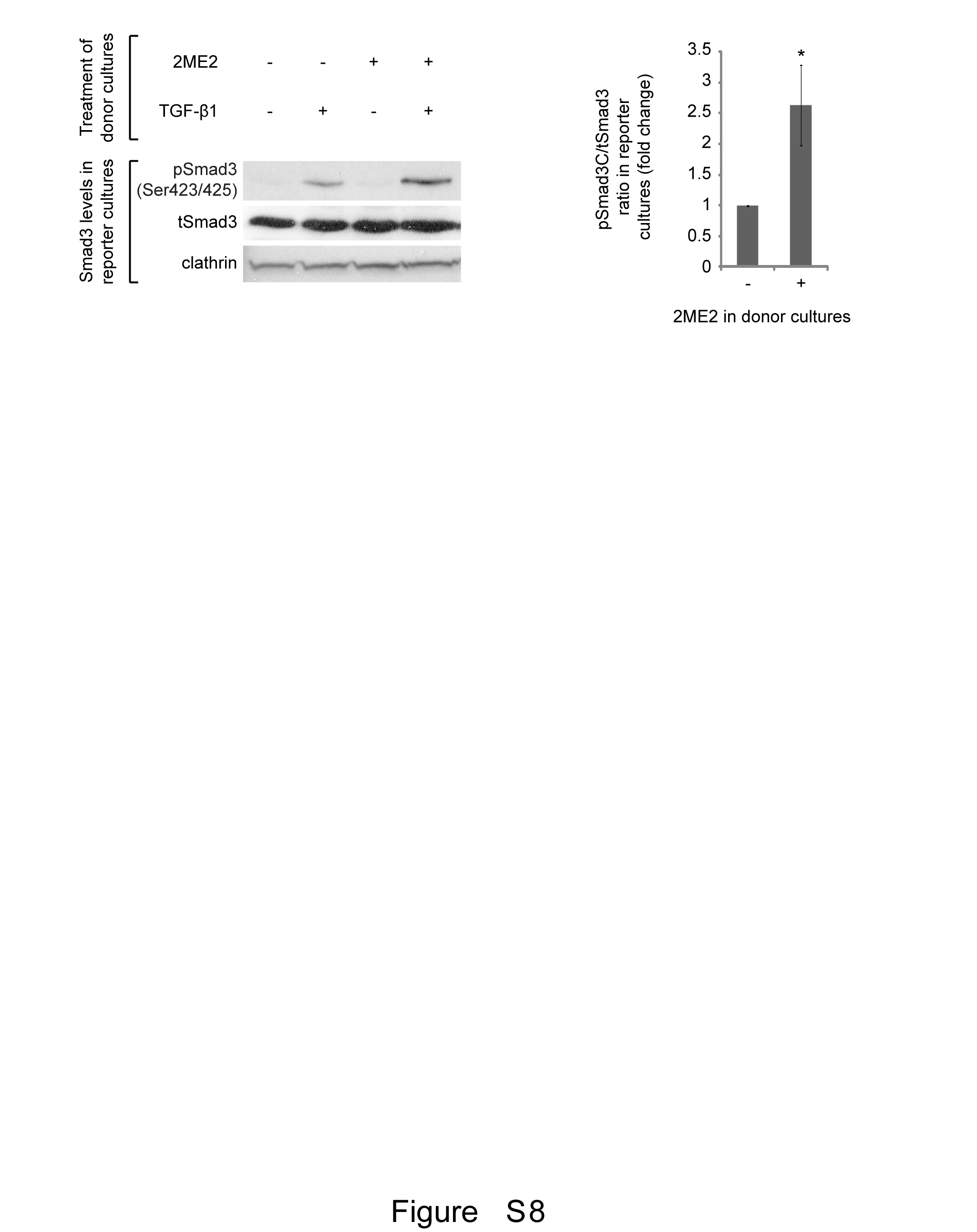

Supplement: Figure S8 — Arrest in mitosis with 2ME2 reduces clearance of TGF-β1 from medium. Cells (referred as “donor cultures”) were arrested in mitosis with 2ME2 or treated with vehicle, and stimulated with TGF-β1 (3 h) or vehicle. Media, collected from these cultures, were transferred to naïve cells (referred as “reporter cultures”), for 1 h of stimulation (as described in Materials and Methods). Panels depict α-pSmad3C, α-tSmad3 and α-clathrin immunoblots of the reporter cultures. Bar graph depicts average ± SEM of the fold increase in pSmad3C/tSmad3 ratio in the lysates of the reporter cultures (media collected from 2ME2-arrested-TGF-β1-stimulated cells, as compared to media collected from non-arrested-TGF-β1-stimulated cells; 2.63±0.65 fold increase, n = 7; *, p<0.023, 1-tailed t-test). (TIF) [file pone.0043459.s008.tif]

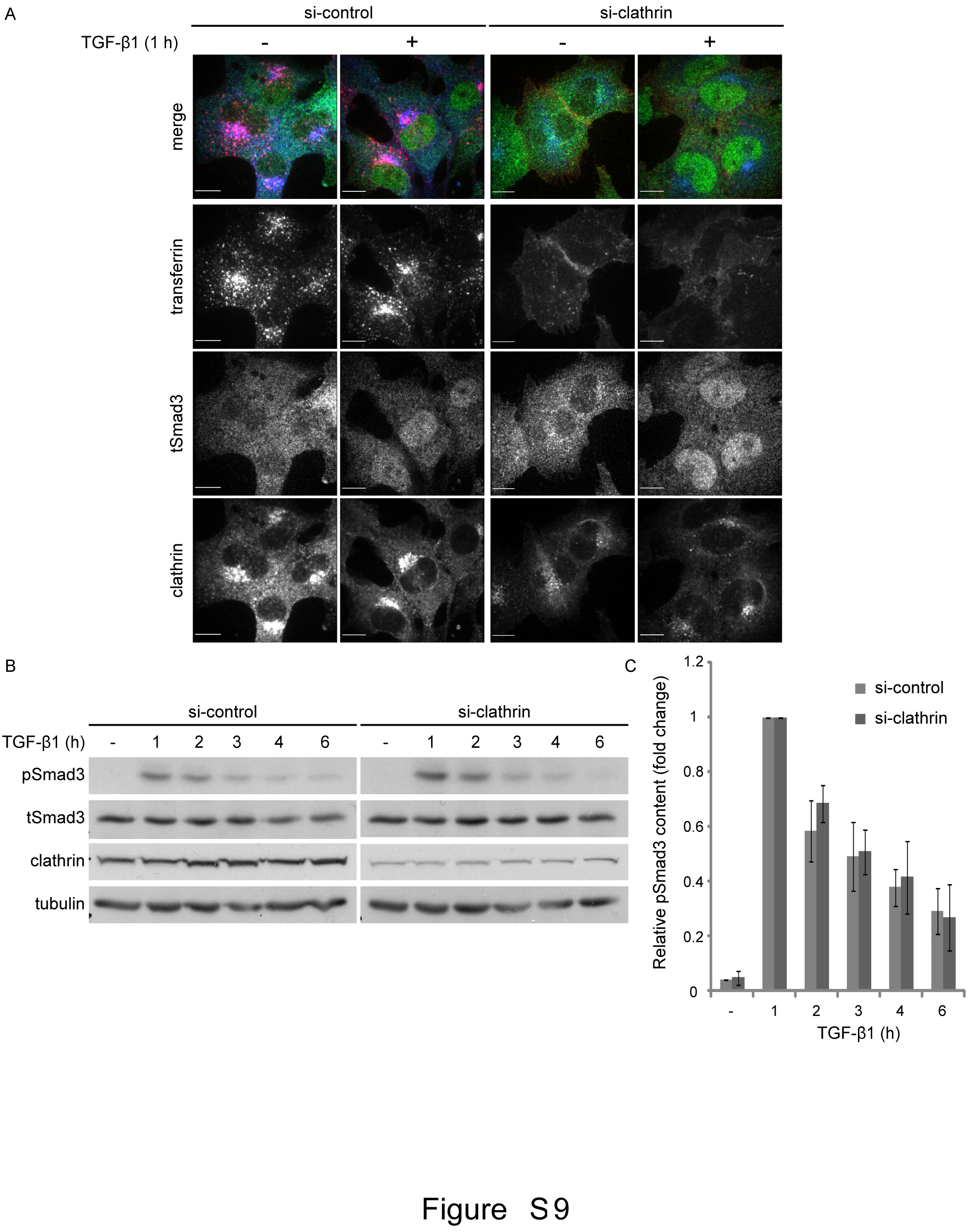

Supplement: Figure S9 — siRNA-mediated depletion of clathrin heavy chain does not block the phosphorylation and nuclear translocation of Smad3 or the attenuation of the TGF-β signal. A, Confocal micrographs of ES-2 cells, transfected with siRNA against clathrin heavy chain or with non-targeting siRNA, activated or not with TGF-β1 (1 h), incubated with fluorescent transferrin (10 min, 100 µg/ml), and stained for clathrin heavy chain and Smad3. B, α-pSmad3C, α-tSmad3, α-clathrin and α-tubulin immunoblot of ES-2 cells, transfected with siRNA against clathrin heavy chain or with non-targeting siRNA and stimulated with TGF-β1 for the indicated times. C, Bar graph depicts the average ± SEM of the pSmad3C/tSmad3/tubulin signal (n = 3, no significant differences were observed between the clathrin depleted and the control cells). (TIF) [file pone.0043459.s009.tif]

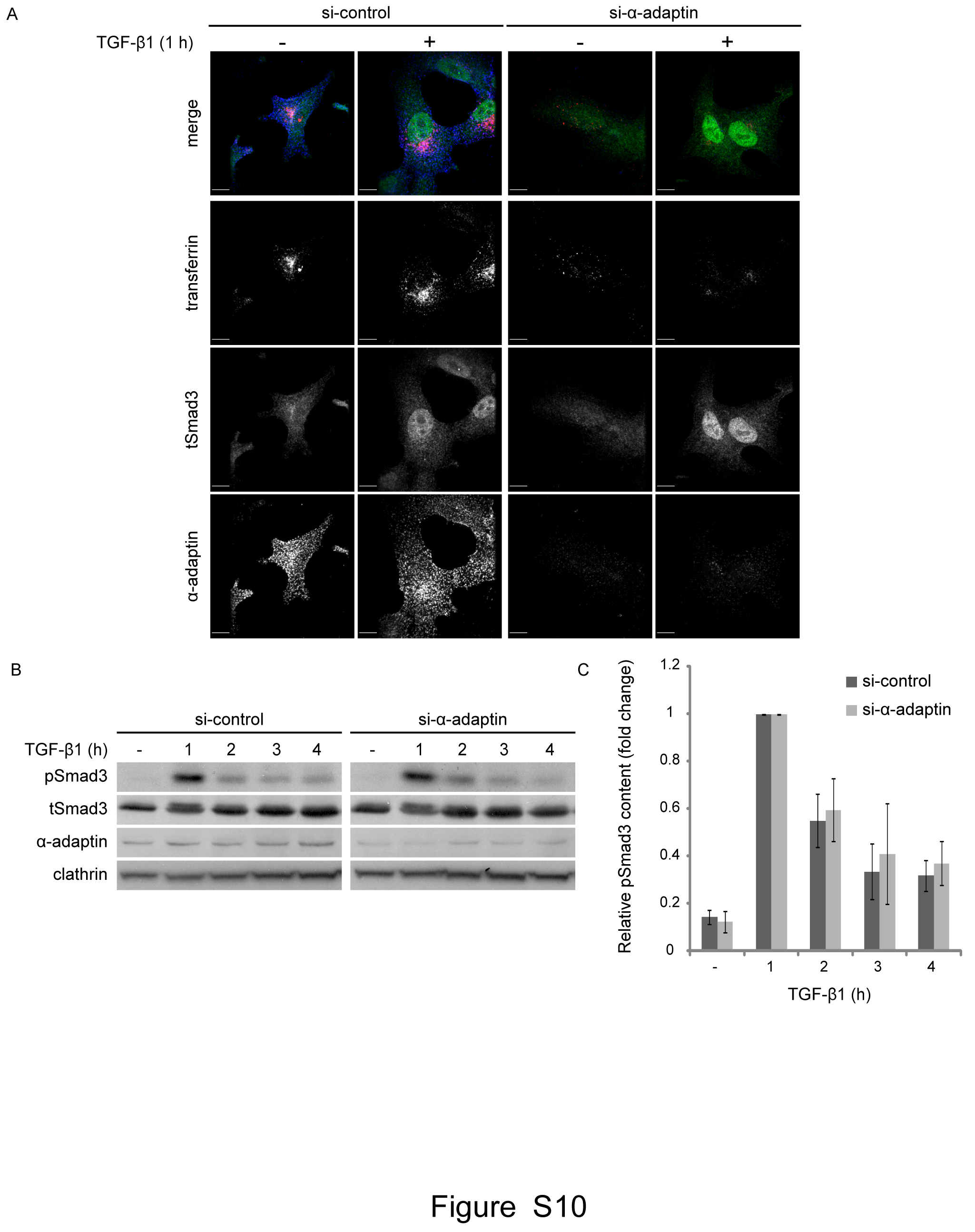

Supplement: Figure S10 — siRNA-mediated depletion of α-adaptin does not block the phosphorylation and nuclear translocation of Smad3 or the attenuation of the TGF-β signal. A, Confocal micrographs of ES-2 cells, transfected with siRNA against α-adaptin or with non-targeting siRNA, activated or not with TGF-β1 (1 h), incubated with fluorescent transferrin (10 min, 100 µg/ml), and stained for α-adaptin and Smad3. B, α-pSmad3C, α-tSmad3 α- α-adaptin, and α-clathrin immunoblot of ES2 cells, transfected with siRNA against α-adaptin or with non-targeting siRNA and stimulated with TGF-β1 for the indicated times. C, Bar graph depicts the average ± SEM of the pSmad3C/tSmad3/tubulin signal (n = 3, no significant differences were observed between the α-adaptin depleted and the control cells). (TIF) [file pone.0043459.s010.tif]

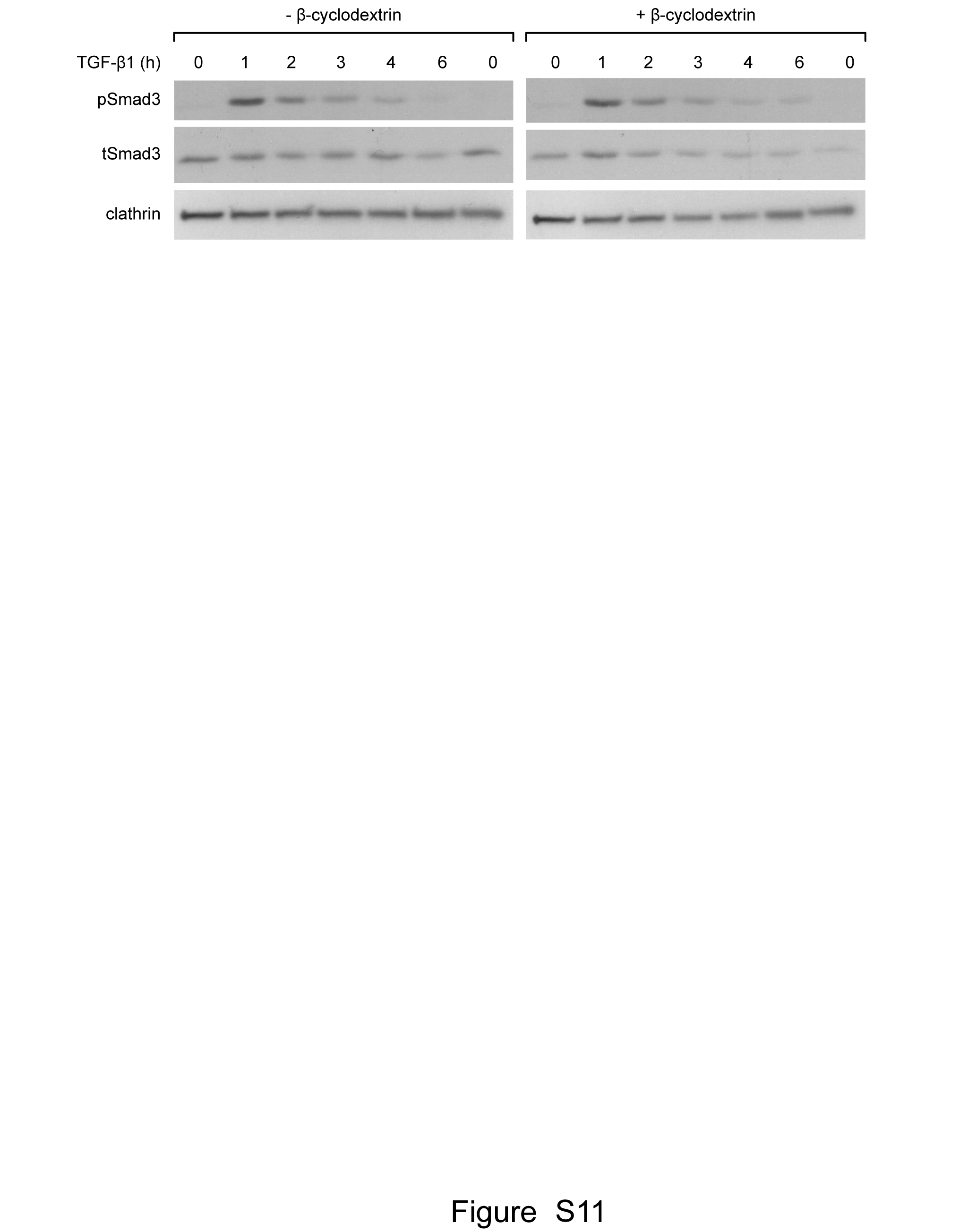

Supplement: Figure S11 — Reduction of cholesterol content does not alter the profile of attenuation of Smad3 phosphorylation. α-pSmad3C, α-tSmad3 and α-clathrin immunoblots of ES-2 cells, treated with β-cyclodextrin (5 mM) or vehicle, and stimulated with TGF-β1 for the indicated times. (TIF) [file pone.0043459.s011.tif]

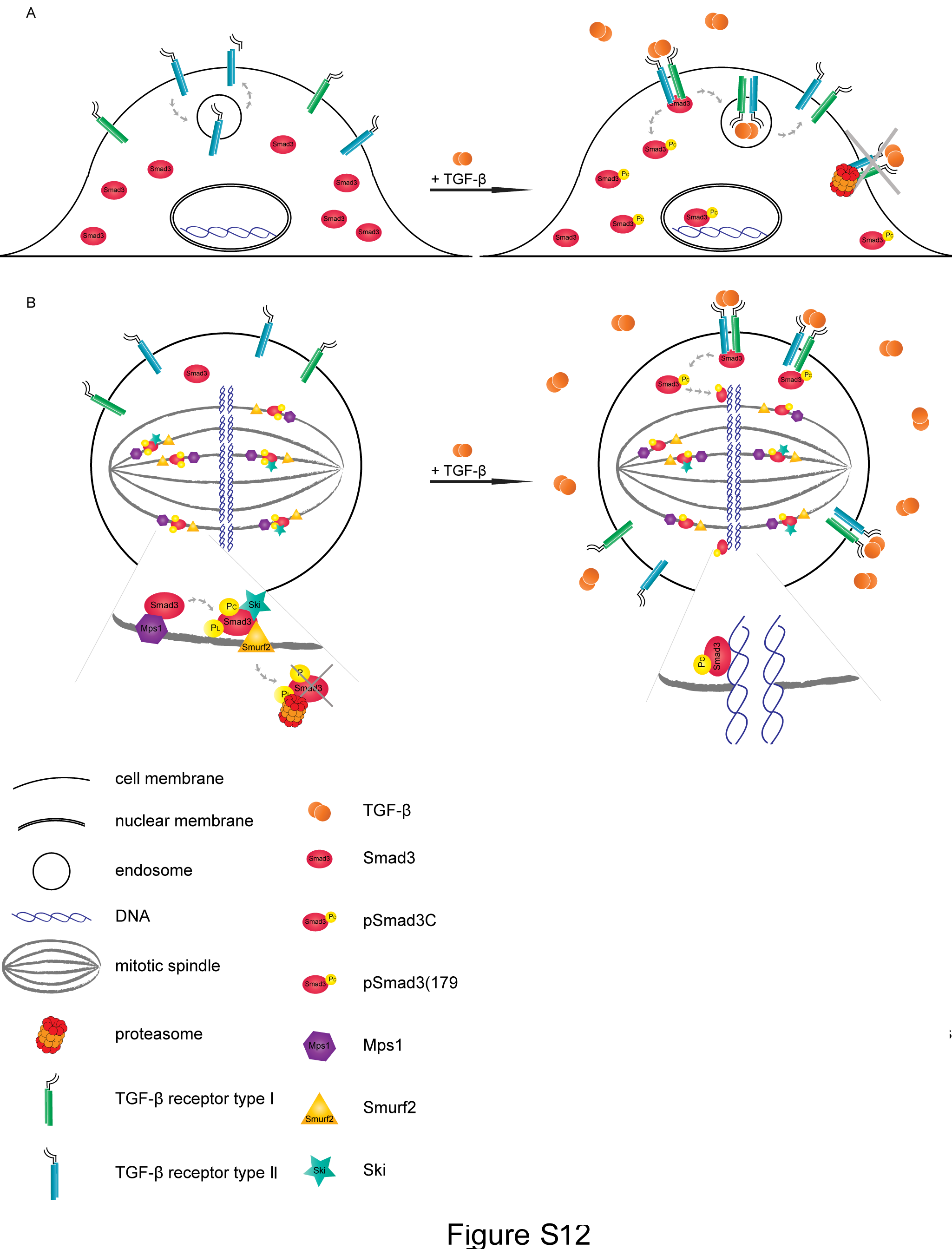

Supplement: Figure S12 — Schematic depiction of the mitosis-induced alterations to Smad3 and TGF-β receptor signaling. A, In interphase cells, TGF-β receptors endocytose and recycle constitutively. Upon TGF-β stimulation, Smad3 is phosphorylated at its C-terminus SSXS motif. Signal attenuation occurs through the proteasome-mediated down-regulation of the signaling receptor complex. B, In un-stimulated mitotic cells, Smad3 is phosphorylated at its C-terminus and threonine 179 in a ligand/receptor -independent fashion and localizes to vicinity of the mitotic spindle. In these conditions, Smad3 binds Smurf2 and Ski and fails to induce a transcriptional response. Moreover, in mitosis a reduction of Smad3 levels is observed. Furthermore, in mitosis, recycling in general and the endocytosis of the TGF-β receptors in particular, are arrested. The endocytosis arrest allows for the maintenance of TGF-β receptors at the plasma membrane. TGF-β-stimulation of mitotic cells induces a transcriptional response through a prolonged phosphorylation of pSmad3C, due to the reduced proteasome-mediated de-activation/degradation of the receptors. (TIF) [file pone.0043459.s012.tif]
